# Multi-cellular human bronchial models exposed to diesel exhaust particles: assessment of inflammation, oxidative stress and macrophage polarization

**DOI:** 10.1186/s12989-018-0256-2

**Published:** 2018-05-02

**Authors:** Jie Ji, Swapna Upadhyay, Xiaomiao Xiong, Maria Malmlöf, Thomas Sandström, Per Gerde, Lena Palmberg

**Affiliations:** 1 Institute of Environmental Medicine, Karolinska Institute, Box 210, SE-171 77 Stockholm, Sweden; 2 Inhalation Sciences Sweden AB, Stockholm, Sweden; 3 Department of Public Health and Clinical Medicine, University Hospital, Umeå, Sweden

**Keywords:** Diesel exhaust particles, Air-liquid interface multicellular model, Inflammation, Macrophage polarization, Oxidative stress

## Abstract

**Background:**

Diesel exhaust particles (DEP) are a major component of outdoor air pollution. DEP mediated pulmonary effects are plausibly linked to inflammatory and oxidative stress response in which macrophages (MQ), epithelial cells and their cell-cell interaction plays a crucial role. Therefore, in this study we aimed at studying the cellular crosstalk between airway epithelial cells with MQ and MQ polarization following exposure to aerosolized DEP by assessing inflammation, oxidative stress, and MQ polarization response markers.

**Method:**

Lung mucosa models including primary bronchial epithelial cells (PBEC) cultured at air-liquid interface (ALI) were co-cultured without (PBEC-ALI) and with MQ (PBEC-ALI/MQ). Cells were exposed to 12.7 μg/cm^2^ aerosolized DEP using Xpose*ALI*^®^. Control (sham) models were exposed to clean air. Cell viability was assessed. CXCL8 and IL-6 were measured in the basal medium by ELISA. The mRNA expression of inflammatory markers (*CXCL8, IL6, TNFα*), oxidative stress (*NFKB, HMOX1, GPx*) and MQ polarization markers (*IL10, IL4, IL13, MRC1, MRC2 RETNLA*, *IL12* and*IL23*) were measured by qRT-PCR. The surface/mRNA expression of TLR2/TLR4 was detected by FACS and qRT-PCR.

**Results:**

In PBEC-ALI exposure to DEP significantly increased the secretion of CXCL8, mRNA expression of inflammatory markers (*CXCL8, TNFα*) and oxidative stress markers (*NFKB, HMOX1, GPx*). However, mRNA expressions of these markers (*CXCL8, IL6, NFKB,* and *HMOX1*) were reduced in PBEC-ALI/MQ models after DEP exposure. TLR2 and TLR4 mRNA expression increased after DEP exposure in PBEC-ALI. The surface expression of TLR2 and TLR4 on PBEC was significantly reduced in sham-exposed PBEC-ALI/MQ compared to PBEC-ALI. After DEP exposure surface expression of TLR2 was increased on PBEC of PBEC-ALI/MQ, while TLR4 was decreased in both models. DEP exposure resulted in similar expression pattern of TLR2/TLR4 on MQ as in PBEC. In PBEC-ALI/MQ, DEP exposure increased the mRNA expression of anti-inflammatory M2 macrophage markers (*IL10, IL4, IL13, MRC1, MRC2*).

**Conclusion:**

The cellular interaction of PBEC with MQ in response to DEP plays a pivotal role for MQ phenotypic alteration towards M2-subtypes, thereby promoting an efficient resolution of the inflammation. Furthermore, this study highlighted the fact that cell–cell interaction using multicellular ALI-models combined with an in vivo-like inhalation exposure system is critical in better mimicking the airway physiology compared with traditional cell culture systems.

**Electronic supplementary material:**

The online version of this article (10.1186/s12989-018-0256-2) contains supplementary material, which is available to authorized users.

## Background

Automobile exhaust mediated air pollution continues to be an unavoidable respiratory health hazard throughout the world. Acute air pollution episodes with high concentrations of particulate matter have been associated with increased emergency room visits and hospitalization due to exacerbation of respiratory diseases like asthma and chronic obstructive pulmonary disease (COPD) [1, 2]. Diesel engines, by virtue of their high efficiency, robustness and low running costs are of wide-usage globally. Diesel exhaust particles (DEP) are the products of incomplete combustion of diesel engine fuel. DEP constitute a complex mixture of particles (< 1.0 μm in diameter) and combustion gases with a carbon core surrounded by trace metals, salts and organic hydrocarbons.

Upon exposure, DEP can deposit on the human airway mucosa. They may cross the epithelium and cell membranes of resident macrophages, subsequently binding to different cytosolic receptors which may lead to cell growth and differentiation [3]. Acute exposure to DEP in humans results in extensive bronchial and alveolar inflammation with influx of phagocytic cells [2, 4]. Long-term exposure to DEP has been associated with greater incidence of cough and chronic bronchitis [5]. Exposure to DEP has also been associated with a number of long-term adverse effects, such as exacerbation of pre-existing lung disease (asthma and COPD), respiratory infections and worsening of cardiovascular disease, as well as increased mortality [6–8].Similarly, many animal studies have examined the effects of DEP and reported alterations in oxidative stress and inflammatory endpoints [9, 10]. Even though animal studies still serve as a major method of mechanistic investigation, current literature underlines several limitations in the study designs particularly related to dosimetry. Large differences in sensitivity between species and within different strains of the same species have also been observed. The response to DEP exposure has been examined in in vitro studies employing airway epithelial cells, nasal epithelial cells, alveolar macrophages, mast cells, and cell lines [11–13]. These studies have shown that DEP can generate reactive oxygen species (ROS), which in turn trigger a variety of cellular consequences, such as DNA damage, apoptosis and inflammatory responses [14, 15]. These potentially injurious effects of ROS can be neutralized by a variety of antioxidants, including Heme oxygenase 1(HMOX1), glutathione peroxidase (GPx), and superoxide dismutase(SOD) [16].

Human airway epithelium acts as the first line of defense between external environment and internal milieu, thus playing a central role in the response to DEP exposure. Airway macrophages (MQ) and epithelial cells are the two most abundant cell types present in both the conducting- and lower airways, thereby serving as the crucial first responders to airborne particles deposited in the lung [17, 18]. Additionally, the airway epithelium acts both as a physical barrier against the inhaled stimulant (DEP) and as an orchestrator of the innate immune response [17, 19].

The classically activated macrophages (M1) arise in response to the T helper type 1 (Th1) cytokine interferon gamma (IFN-γ) and lipopolysaccharide (LPS). M1-MQs possess bactericidal and tumoricidal activity; generate reactive oxygen species (ROS) and nitric oxide; promote Th1 responses; and produce high levels of pro-inflammatory cytokines like tumor necrosis factor alpha (TNF-α) and interleukin (IL-6) etc. [20, 21]*.* On the other hand, the alternative activated macrophages (M2), generated by the T helper type 2 (Th2) cytokines IL-4 or IL-13, play a central role in tissue repair, tissue remodeling, matrix deposition and healing, and promote Th2 responses [20, 21]. M2-MQ express high levels of scavenger mannose and galactose receptors, and produce high levels of IL-10 and IL-1 receptor antagonist [22, 23]. Macrophage polarization have been studied in certain scenarios like bacterial infection, cancer, and asthma [22, 23] and is considered to be an evolving topic of interest. In humans, phenotypic alteration of MQ is considered to play a pivotal role in the onset of airway disease and has potential implications for the treatment of chronic respiratory disease like asthma and COPD.

Byrne et al. [24] reported that M1 associated cytokines; IL-12 and IFN-γ are increased in response to exposure to particulate matter in air pollutants. On the other hand, exposure to cigarette smoke has been shown to alter macrophages towards M2 phenotype [25]. Different nanoparticles can perturb polarization and reprogramming of the macrophages, which is dependent on their chemical composition [26], size [27] and surface coating [28]. According to Miao et al. [29], many nanoparticle types like Ag-NP, Au-NP, ZnO-NP, TiO-NP, and SiO-NP can induce a M1 phenotype polarization and there are a few reports on NP-induced M2-MQ polarizations. The knowledge on DEP exposure related macrophage polarization is still lacking. Based on a study conducted by Jaguin et al. [30] it was shown that by treating human blood monocyte-derived MQ with DEP, the expression of several M1 and M2 markers which are involved in MQ activation was impaired, but without inhibiting the overall polarization process. DEP exposure also attenuated the LPS-induced M1-MQs effects. Because DEP can activate the oxidative stress pathways [31], this may suggest that the alteration of M1/ M2 markers upon DEP exposure is Aryl Hydrocarbon Receptor (AhR)- and Nrf2-dependent. Bauer et al. [24] showed that in co-cultures of human primary alveolar macrophages with epithelial cells, ozone exposure lead to a modified macrophage response inducing M2 activation status with a reduced phagocytic activity. In vivo studies in rats suggest that inhalation of ozone is associated with accumulation of both classically- and alternatively activated MQ in the lungs [32]. It has been well established that close cellular cross talk between airway epithelial cells and MQ in the presence of different stimuli (environmental- or intentional exposure) regulate the inflammatory response in association with macrophage polarization. The lung microenvironment has been shown to influence MQ phenotype- and function [33]. However, most in vitro studies [11, 34] investigating the cellular inflammatory response to air pollutants have used mono-culture systems, which do not address the interaction between different cell types present in the airways, and have limited applicability to in vivo situations. The cross talk between MQ and epithelial cells are essential as they both function within the first line of defense against inhaled toxic agents in both upper- and lower airways.

Previously, we reported that the toll-like receptors (TLR), including TLR2 and TLR4 on the surface of both macrophages [35] and epithelial cells [36], are very critical in recognizing a wide spectrum of inhaled pathogens. Although the involvement of TLR2/4 in the innate immune response to DEP are well known [37], there is still no consensus on how DEP modulate TLR2/4 expression, and especially the interaction of the different cell types involved.

Therefore, in this study we performed DEP exposure with precise dosimetry using air liquid interface (ALI) co-culture models of human primary bronchial epithelial cells (PBEC) with macrophages (THP-1 derived macrophages) to mimic cell-cell interaction of in vivo condition. Further the interaction between epithelial cells and MQ in response to DEP exposure for the alteration of MQ polarization was investigated using both mono- (PBEC-ALI or MQ) and co-cultured (PBEC-ALI/MQ) models.

## Methods

### Cell cultures

#### Bronchial mucosa models with PBEC cultured at ALI (PBEC-ALI)

The PBEC were harvested from healthy bronchial tissues obtained from 10 donors in connection with lobectomy following their informed and written consent. All procedures performed in the study were in accordance to the approval of the Ethical Committee of Karolinska Institutet, Stockholm. The cells used in this study are well characterized and have been used in other studies [36, 38, 39].

The airlifted PBEC models were developed as previously described [39]. Briefly, PBEC were seeded (1 × 10^5^ cell/cm^2^) and cultured on transwell inserts (0.4 μM pore size, BD Falcon™) in twelve-well plates under standard conditions (5% CO_2_ at 37 °C). One ml PneumaCult™-Ex medium (Stemcell technologies, Cambridge, UK) supplemented with 96 μg/ml hydrocortisone (Stemcell technologies, Cambridge, UK) and penicillin streptomycin antibiotics (PEST, 1%, Bio Whittaker, Lonza, Basel, Switzerland) was added to the basal and apical chamber of the insert. In the following 7 days, expand medium was replaced every second day. At confluence (95%), the models were airlifted by aspirating all the PneumaCult™-Ex expand medium and adding 1 ml PneumaCult™-ALI maintenance medium (Stemcell technologies, Cambridge, UK) supplemented with 96 μg/ml hydrocortisone, 2 mg/ml heparin (Stemcell technologies, Cambridge, UK) and 1% PEST to the basal chamber only. Maintenance medium was changed every second day. After 3 weeks of culturing at ALI, the number of PBEC reached 1.5 × 10^6^ cells/insert and the cells were observed in a well differentiated state including ciliated cells and mucus producing cells.

#### THP-1 derived macrophage (MQ)

Human monocyte cell line (THP-1) was purchased from the American Type Culture Collection (TIB-202™, ATCC, Rockville, MD, USA), and grown in T75 flasks using RPMI-1640 cell medium (Gibco Life technologies, Paisley, UK) supplemented with 1% PEST and 10% heat-inactivated fetal bovine serum (FBS; Gibco Life technologies, Paisley, UK) and maintained in 5% CO_2_ at 37 °C. The cell culture medium was replaced every second day. On attainment of the cell concentration 8 × 105 cells/mL, the THP-1 cells were sub-cultured in Petri dishes at a concentration of 1 × 10^6^ cells/ml. To differentiate THP-1 cells into macrophage-like cells [40], 5 ng/ml phorbolmyristate acetate (PMA) (Sigma, Germany) was added in RPMI-1640 cell medium (Gibco Life technologies, Paisley, UK) [41]. After 48 h of incubation, the medium was collected, and the plates were washed three times with PBS. The non-adherent cells were washed away and counted to calculate the adherence rate. In this study, about 70% of the cells were attached and had spread out. The adherent cells were then trypsinized and collected for further analysis. Trypan blue was used to detect the effects of trypsinization. Viability of more than 95% was acceptable. Non-differentiated THP-1 cells and differentiated THP-1 cells were documented using BX50 light microscope (Olympus Optical Co., Tokyo, Japan). Anti-CD68-PE-Cy7 (BD Pharmingen, San Diego, CA, United States) was used as a marker to detect the purity of the MQ by flow cytometry (LSR Fortessa™, BD Bioscience, United States). The determined purity was more than 80% in all cases.

#### Co-culture of PBEC-ALI and MQ (PBEC-ALI/MQ)

In the pilot study, we co-cultured MQ placed underneath the PBEC-ALI models (PBEC-ALI/MQ_sub_), but low effects of the addition of MQ after DEP exposure were seen (Additional file 1: Figure S1, S2). Therefore, in the following studies, PBEC-ALI was co-cultured with MQ on top of the epithelial layer (PBEC-ALI/MQ).

Two hundred μl THP-1 culture medium containing 1.5 × 10^5^ MQ was added to the apical side of the differentiated PBEC models, giving an estimated ratio of MQ: PBEC = 1:10 [42], and was incubated in 5% CO_2_ at 37 °C for 2 h. As a control, mono-culture of MQ were performed by adding 200 μl THP-1 culture medium containing 1.5 × 10^5^ MQ to all inserts. One ml PneumaCult™-ALI maintenance medium with all the supplements was added to the basal chamber of the insert, and incubated in 5% CO_2_ at 37 °C for 2 h. Prior to exposure, after adhesion of MQ, THP-1 culture medium from the apical chamber was aspirated.

### DEP generation and characterization

DEP were generated and collected from a three cylinder, 3.8 l tractor engine (Model 1113 TR; Bolinder-Munktell) at the Swedish Engine Test Center, Uppsala. The engine was run at 1600 rpm on diesel fuel (Swedish environment class MK 3) working at 80% of its rated 41.2 kW output. The exhaust, which was diluted 11-fold with air, was passed through a Tepcon electrostatic filter (Model 2200; Act Air, Cardiff, UK) at a total flow rate of 1600 kg/h and was precipitated on the filter at 44 °C. The DEP soot was scraped from the Teflon-coated electrodes and stored in the dark at − 20 °C.

The aerodynamic particle size distribution of re-aerosolized DEP was analyzed by using a 9-stage Marple Cascade Impactor (MCI, MSP Corp). One mg DEP was loaded to the PreciseInhale™ platform (Inhalation Sciences, Stockholm, Sweden) and generated as aerosols at 100 bar pressure. DEP aerosols were pumped through the MCI at an airflow rate of 2 L/min. Particles were captured by impaction on the MCI stages based on their size. The aerodynamic size distribution of the DEP was then calculated from the amount of particles captured on each stage of the impactor. The experiment was repeated in triplicate.

To characterize the DEP, the particles were deposited on glass cover slips using PreciseInhale exposure system with a cylindrical 1 L holding chamber. The particle deposition flow rate was 120 mL/min. The specimens were mounted on an aluminum stub and sputter coated with 10 nm Platinum (Q150T ES, West Sussex, UK) and analyzed in an Ultra 55 field emission scanning electron microscope (SEM) (Zeiss, Oberkochen, Germany) at 5 kV using the secondary electron detector.

Endotoxin level in the DEP samples was determined using the Limulus amebocyte lysate assay (LAL; Endosafe® Endochrome-K™ U.S. Lisence No.1197, Charles River Laboratories, Wilmington, Massachusetts, USA). According to the manufacturer’s instruction, the particles were diluted in endotoxin specific buffer and *Escherichia coli* 0111: B4 was used as standard.

### Exposure of PBEC-ALI, PBEC-ALI/MQ and MQ to DEP

In order to mimic the in vivo exposure situation of the lung, the model was exposed to clean air (sham) or to aerosolized DEP using the Xpose*ALI* exposure system as previously described [39]. The models including only PBEC (PBEC-ALI), models co-cultured with MQ (PBEC-ALI/MQ) and mono-cultures of MQ were then placed inside the exposure modules. Compressed air of 100 bars was used to aerosolize the DEP into the 300 ml holding chamber. The DEP aerosol was then pulled from holding chamber at a main flow rate of 120 ml/min and diverted into triplicate exposure branches at a flow rate of 10 ml/min. DEP exposures were carried out for 3 mins (the exposure time period was chosen according to pilot study described in Additional file 1). In the corresponding controls, sham exposures were performed with identical flow rate settings- and exposure duration using clean air and a clean system. The inside of the aerosol holding chamber was covered with wet filter papers to maintain the humidity which increased the viability of the cells substantially. In addition, in the exposure module the inserts including bronchial models were always in contact with the basal medium during the exposures.

Following exposure, cells were removed from the exposure modules and placed in 12-well plates with fresh basal medium, and incubated for 24 h in 5% CO_2_ and 37 °C.

### DEP exposure dose and uptake

To calculate the DEP exposure dose and uptake, the models (*n* = 3/time point) were exposed to DEP for 15 s, 45 s and 3mins respectively, corresponding to low-, medium- and high-exposure doses (Additional file 1). After each exposure, DEP were collected from all 3 inserts separately by rinsing them with 200 μL 99% ethanol. The deposited DEP dose in each insert was quantified by measuring the absorbance using spectrophotometric technique (Cary 60 UV-Vis, Agilent Technologies, Palo Alto, CA, United States) [43] and calculating the deposited dose of DEP using a standard curve.

After exposure, a spectrophotometric analysis was performed to quantify the actual exposure dose of DEP in each insert (DI), using the following formula:

$$ DD\left( DEP\  dose\right)=\frac{\mathrm{DI}}{\mathrm{IS}\ \left(\mathrm{insert}\ \mathrm{surface}\right)\ } $$

To investigate the DEP uptake, a commercial Quadri-Wave Lateral Shearing Interferometry (QWLSI) (SID4Bio, Phasics SA, Saint Aubin, France) was directly plugged onto the microscope (Labphot-2, Nikon FX-35DX) to detect distribution of mass across the model.

### Cell viability assay

The cell viability of both PBEC-ALI and PBEC-ALI/MQ were determined after 24 h (DEP versus sham) using three different methods:

- Trypan blue assay: The samples were stained by trypan blue (diluted with PBS in 1:5 ratio) following a 24 h exposure. The viable cell fraction was assessed using conventional light microscopy (Motic, AE2000 Inverted Microscope, Motic Deutschland GmbH, Wetzlar, Germany) using a 20× magnification. Four fields of the insert were selected and in each field 200 cells were counted. The viability of more than 95% was accepted. This assay was repeated twice on separate inserts from each donor.
- Lactate dehydrogenase release (LDH) assay: Cell viability of both PBEC-ALI and PBEC-ALI/MQ were determined by measuring the level of released LDH in the basal media. The assay was carried out according to the manufacturer’s instruction (Thermo Fisher Scientific, Pittsburgh, United States). 50 μl basal medium from sham, DEP exposed cells and LDH positive control (0.2% Triton 100X treated) were transferred to a clear 96-well plate (Nunc, Thermo Fisher Scientific). 50 μl LDH reaction mixture was then added to each sample well and mixed by gentle tapping. After 30 min of incubation at room temperature, the reaction was stopped by adding 50 μl of stop solution. LDH release was quantified by measuring absorbance at 490 nm (A490) and 680 nm (A680) using a plate reader. Data were normalized to sham exposure.
- Annexin V Assay via fluorescence automated cell sorting (FACS): To detect the early and late cellular apoptosis rate of the models after exposure, both PBEC-ALI and PBEC-ALI/MQ after 24 h of incubation post exposure (DEP and sham) were trypsinized and treated with annexin V–PE/7-AAD according to the manufacturer’s instructions (BD Pharmingen, San Diego, CA, United States) [39]. Apoptotic cells were detected by collecting 2000 cells using FACS (LSR Fortessa™, BD Bioscience, United States).

### Quantitative real time polymerase chain reaction (qRT- PCR)

Transcript expression of genes involved in oxidative stress (*NFKB, HMOX1, GPx*), pro-inflammation (*CXCL8, IL6, TNF),* tissue injury/repair (*MMP9* and *TIMP1*) *TLR2/TLR4*and macrophage polarization (M1; *IL23, IL12*, M2; *IL10, IL4, IL13, MRC1, MRC2, RETNLA*) were analyzed using the qRT-PCR technique. The list of genes assessed, and corresponding primer pairs are provided in Additional file 1: Table S1. Total mRNA from both PBEC-ALI and PBEC-ALI/MQ and MQ only were isolated following 24 h exposure (DEP and sham) using the RNeasy Mini Kit (Qiagen; *n* = 6) as described previously [44]. Concentration of RNA was measured using the Nano drop (ND1000 Technology). 1 μg mRNA was reverse transcribed to generate complementary DNA (cDNA) using the high capacity RNA to cDNA kit (Life technologies, Paisley, UK) and a thermal cycler (Mycycler™, Biorad). qRT-PCR was performed using the AB 7500 System. The 20 μl qRT-PCR reaction mix consisted of 10 μl Fast SYBR^®^ Green Master Mix (Life technologies, Paisley, UK), 200 nmol of each primer, 5 ng cDNA, and nuclease free water. Beta actin (*ACTB*) was used as the reference control. Expression of each target gene was quantified as a fold change following normalization with *ACTB* and sham. The results were calculated as 2^-ΔCt^ (ΔCt = Ct (gene of interest) - Ct (beta actin).

### ELISA

Concentrations of IL-6 and CXCL-8 in basal medium were measured using the in-house ELISA method described previously [45]. Commercially available antibody pairs MAB206-15, MAF206-15 and MAB208-15, MAF208-15 (R&D SYSTEMS®, UK) were used to measure IL-6 and CXCL-8 respectively. The detection range was 3-375 pg/ml and 12.5-6400 pg/ml for IL-6 and CXCL-8 respectively. MMP-9, TIMP-1, CC-10, TGF-β, IL-13 and IL-10 in basal medium were measured using purchased DouSet ELISA Kit (DY911, DY970, DY4218, DY240, DY213, DY217B; R&D SYSTEMS^®^, UK). The measurements of TNF-α in basal medium were performed by purchased HS quantikine ELISA Kit (HSTA00E; R&D SYSTEMS®, UK). All the analyses of MMP-9, TIMP-1, TNF-α, CC-10, TGF-β, IL-13 and IL-10 were performed according to the manufacturer. For all the duplicated samples, an intra-assay variation < 10% was accepted.

### FACS

To distinguish between PBEC and MQ in PBEC-ALI/MQ, cells were trypsinized and washed twice with PBS, and incubated with monoclonal antibody (anti-CD68-PE-Cy7; Cat No.25-0689-42, eBioscience, Thermo Fisher Scientific, Pittsburgh, United States). The CD68 ^+^ cells were MQ and CD68 ^−^ cells were PBEC. To analyze the expression of TLR2 and TLR4, the trypsinized cells were incubated with monoclonal antibodies (anti-TLR2-APC, Cat No.558319, BD Pharmingen, San Diego, CA, United States; anti-TLR4-PE, Cat No.12-9917-42, eBioscience, Thermo Fisher Scientific, Pittsburgh, United States) for 30 mins in dark. The cells were then washed 3 times and re-suspended in PBS. For PBEC-ALI, the TLR2/TLR4 were detected directly. For PBEC-ALI/MQ, the cells were gated by anti-CD68 antibody first followed by TLR2/TLR4 detection in both CD68 ^+^ cell populations (MQ) and CD68 ^−^ cell populations (PBEC). For all analysis, unstained cells were used to provide the gating controls for determining positivity [46]. Analyses were performed using the flow cytometer (LSR Fortessa™, BD Bioscienc, United States) and calculated as median fluorescence intensity (MFI).

### Statistics

The results are expressed as medians and interquartile ranges (25^th^-75^th^ percentiles). All the comparisons between groups were performed by Wilcoxon signed rank t test. A *p*-value < 0.05was considered as significant. All the data were analyzed using the STATISTICA9 (StatSoft, Inc. Uppsala, Sweden) software.

## Results

### DEP characterization

**Fig. 1:**
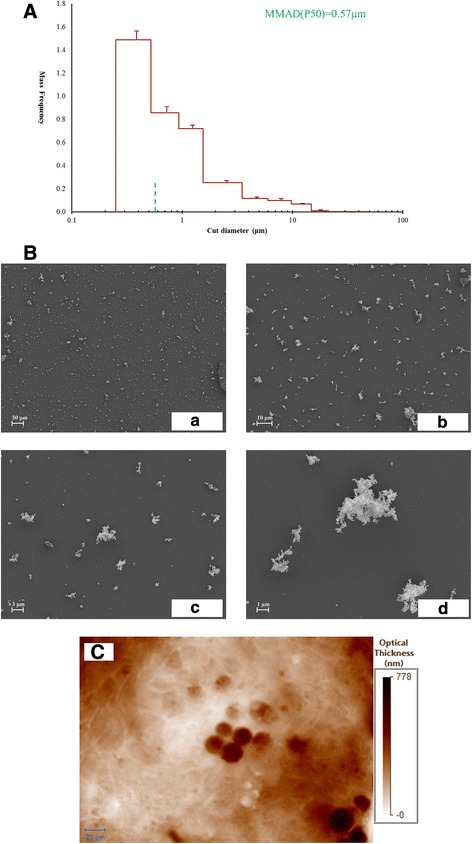
Characterization and uptake of diesel exhaust particles (DEP) (**A**-**C**). **A**: Mass distribution of DEP in different size ranges measured by the 9-stage Marple Cascade Impactor; MMAD (P50): Mass Median Aerodynamic Diameter; The MMAD (P50) of DEP was estimated to be 0.57 μm. 1B: SEM image of DEP with different magnifications; **B** (a): 500X, bar: 30 μm; **B** (b): 2000X, bar: 10 μm; **B** (c): 5000X, bar: 3 μm; **B** (d): 15000X, bar: 1 μm. **C**: Quantitative phase image of the apical side of PBEC-ALI/MQ, which reflects the distribution of mass across the field; Bar: 20 μm

The re-suspended DEP aerosols used in this study had a particle size distribution as shown in Fig. 1A. The mass median aerodynamic diameter (MMAD, P50) was calculated to be 0.57 μm, which may represent a slight overestimation of the actual MMAD because the major fraction of particles was deposited on the cascade impactor end filter without further size separation. Figure 1b indicated SEM analysis of DEP at different magnifications. Figure 1B (a) showed that DEP were evenly distributed over the surface area of exposure with Xpose*ALI* exposure system. Figure 1B (d), high resolution SEM image elucidates the agglomerated structure of DEP, which is in agreement with a previous report by Kireeva et al. [47]. According to the LAL test, the DEP are free from LPS contamination (data not shown).

### DEP exposure: Dose and uptake

According to spectrophotometric analysis the dose per surface area was:

$$ DD=\frac{57.17\mu g/ ml\times 0.2 ml}{0.9\  cm2}=12.7\mu g/c{m}^2 $$

Based on Fig. 1C, the MQ appeared more contrasted, which made them easy to be distinguished among the epithelial cells after DEP exposure. Some parts of MQ were very dense which may indicate the engulfment of DEP particles. Also MQ can be segmented in order to measure the mass of each of them. Based on these measurement (data not shown), MQ mass were higher than the surface mass of epithelial area, which may confirm phagocytosis of DEP particles.

### Effects of DEP on cell viability

Based on the trypan blue staining procedure a cell viability more than 95% was accepted. The results of the colorimetric LDH assay on both PBEC-ALI and PBEC-ALI/MQ models are shown in Additional file 1: Figure S3A. The early apoptosis rate was between 4% and 22.5%; the late apoptosis rate was between 0.2% and 8.5%, and the total apoptosis rate was between 5.5% and 25%, when assessed with annexin V–PE/7-AAD 24 h, following exposure to DEP (Additional file 1: Figure S3B). Trypan blue staining, LDH activity assay and apoptosis assay did not exhibit any alteration before and after exposure to DEP. Thus, none of the DEP doses used in this study were cytotoxic.

### Effects of DEP exposures in mono-culture models compared with co-cultured models

#### Pro-inflammatory, oxidative stress and tissue injury/repair responses

**Fig. 2:**
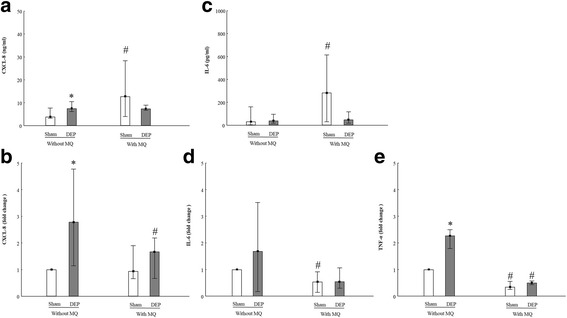
Release and mRNA expression of inflammatory biomarkers in models after exposure to diesel exhaust particles (DEP). Levels of CXCL-8 (**a**) and IL-6 (**c**) secretion in basal medium in PBEC-ALI and PBEC-AL/MQ (*N* = 9) after exposure to DEP and incubated for 24 h; Fold change of *CXCL8* (**b**), *IL6* (**d**) and *TNFα* (**e**) expression in PBEC-ALI and PBEC-AL/MQ(*N* = 6) after exposure to DEP and incubated for 24 h; Exposure: sham: clean air; DEP: 12.7 μg/cm^2^; Data presented as median and 25^th^ -75^th^percentiles, fold change =2^-ΔCt^ of models / 2^-ΔCt^ of sham exposed PBEC-ALI; *: *P* < 0.05 VS Sham exposure; #: *P* < 0.05 VS PBEC-ALI

One of the key features of DEP-associated health effects is inflammation; Secretion and mRNA expression of CXCL-8 (Fig. 2a, b) and IL-6 (Fig. 2c, d) as well as mRNA expression of *TNFα* (Fig. 2e) were detected. We also found that the secretion of CXCL-8 was significantly induced by the DEP exposure in PBEC-ALI (Fig. 2a). An increased mRNA expression of *CXCL8* (Fig. 2b: 3-fold,) and *TNFα* (Fig. 2e: 2.5-fold) was detected. Both CXCL-8 and IL-6 secretion were significantly increased after sham exposure in PBEC-ALI/MQ compared to the PBEC-ALI (Fig. 2a, c). Interestingly, *IL6* and *TNFα* transcript after sham exposure (Fig. 2d, e) as well as *CXCL8* and *TNFα* transcript after DEP exposure (Fig. 2b, e) were significantly decreased in PBEC-ALI/MQ compared to PBEC-ALI.

**Fig. 3:**
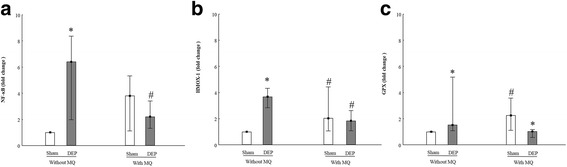
mRNA expression of oxidative stress related markers in models after exposure to diesel exhaust particles (DEP). Fold change of *NFKB* (**a**), *HMOX1* (**b**) and *GPx* (**c**) expression in PBEC-ALI and PBEC-AL/MQ (N = 6) after exposure to DEP and incubated for 24 h; Exposure: sham: clean air; DEP: 12.7 μg/cm^2^; Data presented as median and 25^th^ -75^th^percentiles, fold change =2^-ΔCt^ of models / 2^-ΔCt^ of sham exposed PBEC-ALI; *: *P* < 0.05 VS Sham exposure; #: *P* < 0.05 VS PBEC-ALI

Oxidative stress is also considered as a main effect of DEP-induced toxicity. Redox sensitive transcription factors like *NFKB*, antioxidant enzymes like *HMOX1* and *GPx* are considered to play important roles in this process. In PBEC-ALI, exposure to DEP increased the mRNA expression of *NFKB* (> 6-fold) (Fig. 3a), *HMOX1* (4-fold) (Fig. 3b) and *GPx* (1.5-fold) (Fig. 3c). However, compared to sham exposure, DEP exposure significantly reduced *GPx* mRNA expression in PBEC-ALI/MQ (Fig. 3c). In sham exposed models, *HMOX1* (Fig. 3b) and *GPx* (Fig. 3c) mRNA expressions were twice as high in PBEC-ALI/MQ than in PBEC-ALI. However, after DEP exposure, *NFKB* (Fig. 3a) and *HMOX1* (Fig. 3b) mRNA expressions were significantly lower in PBEC-ALI/MQ than in PBEC-ALI.

**Fig. 4:**
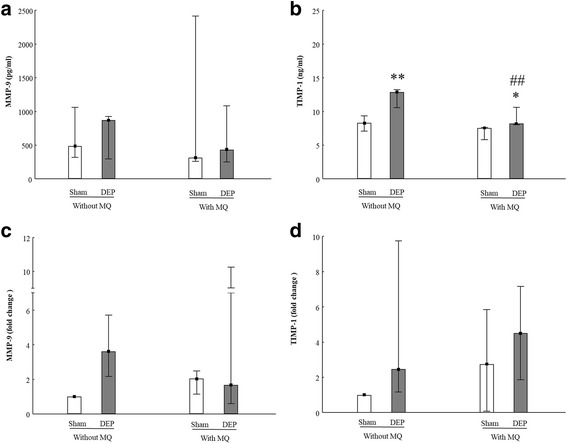
Release and mRNA expression of extra-cellular markers in models after exposure to diesel exhaust particles (DEP). Levels of MMP-9 (**a**) and TIMP-1 (**b**) secretion in basal medium in PBEC-ALI and PBEC-AL/MQ (N = 9) after exposure to DEP and incubated for 24 h; Fold change of MMP-9 (**c**) and TIMP-1 (**d**) expression in PBEC-ALI and PBEC-ALI/MQ (N = 6) after exposure to DEP and incubated for 24 h; Exposure: sham: clean air; DEP: 12.7 μg/cm^2^; Data presented as median and 25^th^ -75^th^percentiles,fold change =2^-ΔCt^ of models / 2^-ΔCt^ of sham exposed PBEC-ALI; *,**: *P* < 0.05, 0.01 VS Sham exposure; ##: *P* < 0.01 VS PBEC-ALI

Pro-inflammatory cytokines and oxidative stress induced by macrophages or epithelial cells may contribute to DEP-induced epithelial damage. In the present study, both MMP-9 and TIMP-1 were detected at both protein- and mRNA levels (Fig. 4a-d). We found that DEP exposure increased TIMP-1 secretion in both models (Fig. 4b). After DEP exposure, PBEC-ALI/MQ released lower levels of TIMP-1 than PBEC-ALI (Fig. 4b). The secretion of MMP-9 was not altered in PBEC-ALI or PBEC-ALI/MQ, but an increased secretion of MMP-9 from DEP exposure when culturing MQ only was observed (*P* = 0.027, data not shown). No effect on expression of MMP-9 and TIMP-1 were detected in either PBEC-ALI or PBEC-ALI/MQ models. In regard to TNF-α, TGF-β and IL-13 secretions, most of the samples were below detection limit, and for CC-10 and IL-10, no significant difference was found (data not shown).

#### TLR expression

**Fig. 5:**
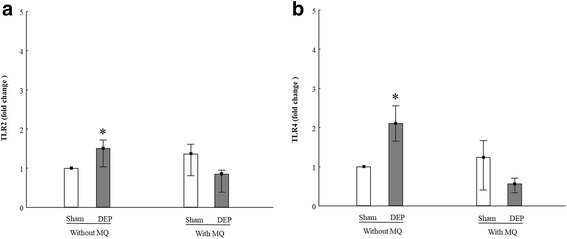
mRNA expression of Toll-like receptors in models after exposure to diesel exhaust particles (DEP). Fold change of TLR2 (**a**) and TLR4 (**b**) expression in PBEC-ALI and PBEC-AL/MQ (N = 6) after exposure to DEP and incubated for 24 h; Exposure: sham: clean air; DEP: 12.7 μg/cm^2^; Data presented as median and 25^th^ -75^th^ percentiles, fold change =2^-ΔCt^ of models / 2^-ΔCt^ of sham exposed PBEC-ALI; *: *P* < 0.05 VS Sham exposure

**Fig. 6:**
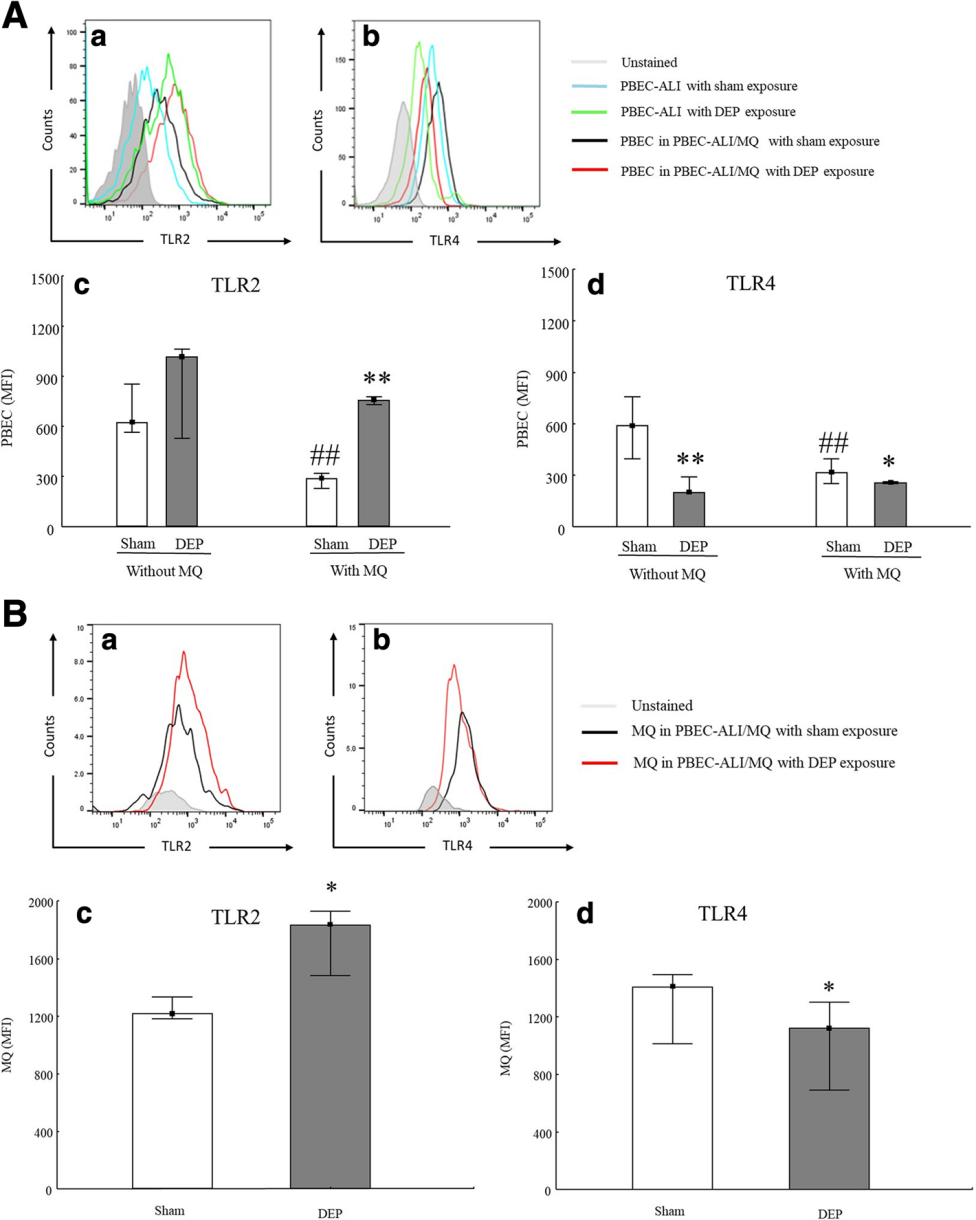
TLR2 and TLR4 expression on the surface of primary bronchial epithelial cells (PBEC) and macrophages (MQ) after exposure to diesel exhaust particles (DEP). PBEC (**A**) and MQ (**B**) were identified by anti-CD 68-PE-Cy7. A representative mean fluorescence intensity (MFI) of 9 experiments is shown (**A** (a&b), **B** (a&b)); the expression of TLR2 (**A** (c)) and TLR4 (**A** (d)) on surface of PBEC in PBEC-ALI and PBEC-AL/MQ was presented as median and 25^th^ -75^th^ percentiles (N = 9); *, **: *P* < 0.05, 0.01 VS Sham exposure; ##: P < 0.01 VS PBEC-ALI. The expression of TLR2 (**B** (c)) and TLR4 (**B** (d)) on surface of MQ which have been co-cultured with PBEC was presented as median and 25^th^ -75^th^ percentiles (N = 9); *: P < 0.05 VS Sham exposure; Exposure: sham: clean air; DEP: 12.7 μg/cm^2^

To clarify the impact of DEP exposure on TLRs expression within mono- and co-culture settings, we detected both mRNA expression and cell surface expression of *TLR2* and *TLR4* by using qRT-PCR and FACS analysis, respectively. The mRNA expression of *TLR2* and *TLR4* were increased by DEP exposure in PBEC-ALI, while unchanged in PBEC-ALI/MQ (Fig. 5). For PBEC-ALI, TLR2 and TLR4, surface expression on PBEC were detected by FACS using anti-TLR2/4 antibodies directly. But for PBEC-ALI/MQ, anti-CD68 antibody was used to distinguish between PBEC (CD68 ^−^) and MQ (CD68 ^+^) using FACS (Additional file 1: Figure S4). The ratio of CD68^+^ and CD68^−^ cells was 1:10, indicating that the ratio between MQ and PBEC was 1:10, which in turn matched the ratio we seeded (MQ: PBEC = 1:10). After CD68 gating, the TLR2/4 surface expression on PBEC or MQ could be detected separately. After sham exposure, the expression of both TLR2 and TLR4 on PBEC in PBEC-ALI/MQ was significantly attenuated compared to PBEC-ALI (Fig. 6A (c, d)). Figure 6A (c) revealed that following DEP exposure, an increase of TLR2 expression on PBEC surface in PBEC-ALI/MQ was detected. However, DEP exposure significantly reduced the surface expression of TLR4 on PBEC in both PBEC-ALI and PBEC-ALI/MQ (Fig. 6A (d)). DEP exposure resulted in a similar surface expression pattern with increased TLR2 and decreased TLR4 expression on MQ in PBEC-ALI/MQ (Fig. 6B (c, d)). For MQ mono-culture, there was no difference between sham and DEP exposure regarding TLR2/TLR4 surface expression (data not shown). Further, there was no difference of TLR2/TLR4 surface expression between MQ mono-culture and MQ in PBEC-ALI/MQ (data not shown).

#### MQ polarization

**Fig. 7:**
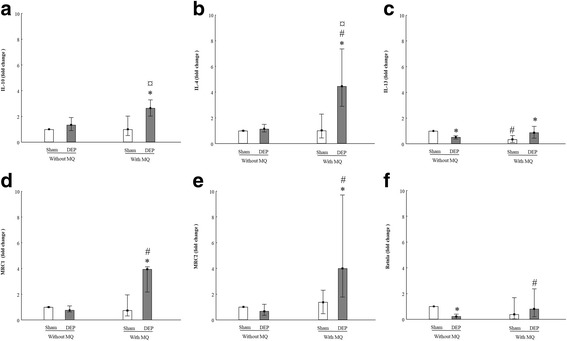
mRNA expression of M2 macrophage markers after exposure to diesel exhaust particles (DEP). Fold change of *IL10* (**a**), *IL4* (**b**), *IL13* (**c**), *MRC1* (**d**), *MRC2* (**e**) and *RETLNA* (**f**) expression in PBEC-ALI and PBEC-AL/MQ (N = 6) after exposure to DEP and incubated for 24 h; Exposure: sham: clean air; DEP: 12.7 μg/cm^2^; Data presented as median and 25^th^ -75^th^ percentiles, fold change =2^-ΔCt^ of models / 2^-ΔCt^ of sham exposed PBEC-ALI; *: *P* < 0.05 VS Sham exposure; #: *P* < 0.05 VS PBEC-ALI; ¤: *P* < 0.05 VS Sham exposed PBEC-ALI

As polarization is a critical step in macrophage activation, we detected the mRNA expression of specific M1 (*IL23* and *IL12*) and M2 (*IL10*, *IL4*, *IL13*, *MRC1*, *MRC2* and *RETNLA*) macrophage markers in both models to elucidate the effects of both DEP exposure and co-culturing of MQ with PBEC and its effects on polarization. DEP exposure increased all M2 macrophage transcription markers in PBEC-ALI/MQ, except *RETNLA* (Fig. 7f). However, with PBEC-ALI, there was no induction by DEP exposure on M2 macrophage markers or even a reduced effect by DEP on *IL13* (Fig. 7c) and *RETNLA* (Fig. 7f) mRNA expression. After DEP exposure, *IL4* (Fig. 7b), *MCR1* (Fig. 7d), *MRC2* (Fig. 7e), and *RETNLA* (Fig. 7f) mRNA expression were all increased in PBEC-ALI/MQ models compared to PBEC-ALI. There was no such increase observed after sham exposure. As for M1 macrophage markers, there was no statistically significant change between the different models tested (Additional file 1: Figure S5). Interestingly, when mono-culture with MQ only were exposed to DEP, there was no significant influence on either M1 or M2 macrophages markers (data not shown).

## Discussion

Our experimental set up to assess the pulmonary toxicity of DEP using co-cultured models consisting of both PBEC and MQ under ALI condition (PBEC-ALI/MQ), and delivery of dry aerosolized particles using the Xpose*ALI*^*®*^ system with precise dosimetry, offers several unique advantages over the existing models and particle delivery systems. Most of the reported studies have used the conventional mono-culture or co-culture models with lung cells (cell lines or primary cells) under submerged conditions, where the particles have been added directly to the cell culture medium [12–14]. Submerged cell culture conditions and addition of stimulants directly to the cell culture media does not reflect the physiology of airway mucosa (consisting of more than 40 cell types [48]) and inhalation exposure conditions [49, 50]. Moreover, it has been well established that the addition of particles directly to the cell culture medium increases the possibility of particle agglomeration which often leads to unreliable outcomes and therefore limit the reproducibility of the system [51, 52]. We have previously demonstrated that our PBEC-ALI model contains ciliated cells, goblet cells, basal cells and club cells, thereby efficiently mimicking the human bronchial mucosa [7]. THP-1 derived macrophages, identified using the FACS methodology, were then added to the apical side of PBEC-ALI to study cell-cell cross talk and corresponding molecular signaling. Realistic inhalation exposure scenarios were achieved through portion by portion aerosolization of DEP from dry powder formulation using compressed air [15, 16]. The use of high-pressure energy resulted in a high degree of deagglomeration into finer aerosols. The preheated Xpose*ALI*^*®*^ system and humidified fine aerosols then reached the cell surface with homogenous distribution, thereby mimicking a real-life inhalation exposure. The multi-cellular bronchial mucosa models with PBEC and THP-1 derived macrophages cultured at ALI, in combination with advanced Xpose*ALI*^*®*^ system used in this study, therefore offers a more realistic and physiologically relevant inhalation exposure scenario with a reliable dose delivery system.

Inflammatory and oxidative stress response following exposure of PBEC-ALI to DEP is evident from the increased secretion of *CXCL8* and *TIMP1*, and increased mRNA expression of *CXCL8, TNFα, NFKB, HMOX1* and *GPx*. Some of these effects were attenuated in the presence of MQ in PBEC-ALI/MQ. *TIMP1* is an inhibitor of Matrix metallopeptidase 9 (*MMP9*), which plays an important role for variety of homeostatic functions and elicit repair responses as balance mechanisms in many chronic lung diseases like COPD [53]. Hence, increased *TIMP1* secretion indicated effects on ECM regulation. Previous studies [12, 54, 55], reported that following exposure to DEP there was an increased release of pro-inflammatory markers, like CXCL-8, IL-6 and TNF-α, both in an ALI model containing human alveolar epithelial tissue models and in submerged models of epithelial cell lines. Our findings are consistent with previous reports. It has been shown by many researchers that exposure to DEP may induce ROS production [56]. Increased ROS may cause the translocation of *NFR2* into the nucleus [57] and binding to antioxidant response elements. Subsequently, antioxidant genes like *HMOX1* or *GPx* [58] can be activated. In line with those studies [54, 59], DEP exposure induced an increased expression of *NFKB*, *HMOX1* and *GPx* in PBEC-ALI models. Interestingly, in PBEC-ALI/MQ, which are PBEC-ALI models co-cultured with THP-1 derived macrophages, the expression of inflammatory genes (*CXCL8* and *TNFα*), antioxidant gene (*HMOX1*) and *NFKB*, as well as TIMP-1 secretion were reduced compared with PBEC-ALI. In contrast, Ishii *et el* [60] reported an increased inflammatory response in submerged co-culture models consisting of PBEC and alveolar macrophages after exposure to ambient particulate matters (PM). Similarly, *TIMP1* expression remained unaltered in A549 and 16HBE cells following exposure to DEP under submerged conditions [61, 62]. This observed difference in inflammatory- and oxidative stress response between our study and those of others [60–62] may be caused not only by the use of different in vitro models (ALI versus submerged culture conditions) or different cell type, but also by the chosen exposure model, where the PBEC-ALI/MQ models were exposed to aerosolized DEP using the Xpose*ALI* system. The exposure of PBEC-ALI/MQ models to aerosolized DEP allows the direct study of cell-particle interaction in contrast to particle exposures performed under submerged conditions, which may lead to the excessive agglomeration of the study particles [50, 51]. Further, the direct interaction of aerosolized particle with cells (DEP with PBEC-ALI/MQ) may initiate faster phagocytosis of particles by MQ, and subsequent release of various pro-inflammatory and anti-inflammatory mediators/cytokines, which in turn may play a crucial role in both innate and adaptive immunity to prevent unnecessary inflammatory response to stimulants [63].

To investigate the possible modulation of TLR signaling by DEP exposure, we analyzed both cell surface and mRNA expression of TLR2 and TLR4. CD68 antibody was used to distinguish between the cell surface expression on PBEC and MQ in PBEC-ALI/MQ models. In this study, both PBEC and MQ in PBEC–ALI/MQ showed an increased surface expression of TLR2, while TLR4 was decreased after DEP exposure. These changes after DEP exposure were not observed in either mono-culture models of PBEC or MQ, with exception of TLR4 in PBEC-ALI. On the other hand, exposure to DEP increased mRNA expression of both TLR2 and TLR4 in PBEC-ALI. No significant change was observed in PBEC-ALI/MQ regarding TLR2 or TLR4. In contrast to results detected using FACS, where surface expression on PBEC and MQ could be separated, the mRNA expression in PBEC-ALI/MQ included total expression of both PBEC and MQ. To our knowledge, this is the first study using FACS analysis showing the difference in cell surface expression of TLR2 and TLR4 among multicellular models including both PBEC and MQ after exposure to DEP. According to a previous study [64], alveolar macrophages treated with PM containing low levels of endotoxin induced a TLR2-dependent pathway, which led to the upregulation of TLR2 but a downregulation of TLR4 mRNA expression. As a comparison, exposure to PM with high levels of endotoxin, a TLR4-dependent pathway was activated. However, in contrast to this study with alveolar macrophages, another study using epithelial cells [65] showed contrasting results, where TLR2 expression was not altered after exposure to PM while TLR4 expression increased by PM exposure. Williams et al. [66] demonstrated a downregulation of both TLR2 and TLR4 expression in human myeloid DCs exposed to PM. Therefore, it seems that different cell types and the interaction between them may influence the surface expression of TLR2 and TLR4. Moreover, since endotoxin levels were below detection limit in the DEP used, it is reasonable to assume that, under co-culture condition, DEP mainly activated and modulated TLR2 signaling, which induced an increased cell surface expression of TLR2.

We found that after sham exposure, the surface expressions of TLR2 and TLR4 on PBEC were decreased when co-cultured with MQ, while there were no such alternations after DEP exposure. Interestingly, regardless of DEP exposure there was no difference between surface expression of TLR2/TLR4 on MQ in PBEC-ALI/MQ and MQ mono-culture. Since macrophages have been demonstrated to express at least 10 times more TLR4 than epithelial cells [65], it is possible that during co-culture, MQ take the place of PBEC regarding host defense and decrease the sensitivity of PBEC by inducing a down-regulated expression of TLRs on PBEC. But this protection effect may be masked by strong stimuli like DEP. As a response to particles different cell types expressed different TLRs and the expression level of each TLRs varies a lot between cell types. DEP exposure induced mRNA expressions of both TLR2 and TLR4 in PBEC-ALI, while there was no such increase in PBEC-ALI/MQ. When ligands such as DEP are bound to TLR2 and TLR4, MyD88- or TRIF, pathways may be activated which may lead to *NFKB-* or activator protein (*AP1*) activation [67]. After translocation into the nucleus, these transcription factors subsequently induce the expression of inflammatory genes. Therefore, this may also explain why DEP exposure increased CXCL-8 secretion/mRNA expression and *TNFα* mRNA expression in PBEC-ALI, but not in PBEC-ALI/MQ. But in order to clarify the mechanisms and reveal the implications of DEP exposure regarding TLR pathways, more detailed studies are needed.

It has been shown that through TLRs signaling, bronchial epithelial cells can regulate the inflammatory response of immune cells like macrophages [68]. Polarization is a key feature of macrophages as a result of stimulation, and two major polarization states have been described. Classically active type1 (M1) macrophages exert pro-inflammatory activities by releasing cytokines like TNF-α and IL-6 [69], and alternatively activated type 2 (M2) macrophages secrete anti-inflammatory cytokines like IL-10 with limited production of pro- inflammatory cytokines [70]. Therefore, the reduced expression of pro-inflammatory markers in PBEC-ALI/MQ models compared with PEBC-ALI, may be due to the polarization of macrophages to M2 phenotype, leading to increased secretion of anti-inflammatory mediators. To confirm our hypothesis, we analyzed markers of both M1 and M2 phenotypes and detected the expression of inflammatory (*TNFα*, *IL6, CXCL8)* anti-inflammatory genes (*IL10, IL4* and *IL13*) as well as typical M2 genes (*MRC1*, *MRC2* and *RETNLA*) [69, 71]. In PBEC-ALI/MQ, the transcription of anti-inflammatory genes or M2 genes like *MRC1* and *MRC2* expression were increased after DEP exposure compared with sham exposure. DEP-induced effects on MRC1 and MRC2 were not detected in PBEC-ALI models. Moreover, DEP exposure led to an up-regulation of other M2 genes (*IL10, IL4, IL13, RETNL*) in PBEC-ALI/MQ compared to PBEC-ALI. In sham exposed models no such up-regulation were observed, either in co-cultured models or in mono- culture, or whether these cultures included PBEC or MQ. However, in PBEC-ALI models, DEP exposure did not increase *IL10* or *IL4* expression, and even decreased *IL13* expression. Therefore, the anti-inflammatory effects of DEP exposure that only existed in co-culture models depended on a cross-talk between PBEC and MQ, which is also evident from several other studies [17, 63].

Further, the polarization process of macrophage has been shown to be closely associated with different levels of TLR expression on its surface. According to Sauer et al. and Orr et al. [72, 73], the ratio of TLR4 and TLR2 was higher in M1-MQs than in M2-MQs, and the TLR4 deficiency can promote the activation of M2-MQs. These findings were in line with our results that the TLR4 surface expression were downregulated in MQ of PBEC-ALI/MQ after DEP exposure. NF-κB which is an end point and key master of TLR pathways can also regulate the MQ polarization [74]. Similarly, we showed that in PBEC-ALI, DEP exposure induced NF-κB activation and promoted inflammatory effects by upregulation of *CXCL8* and *TNFα*. However, in PBEC-ALI/MQ, macrophages were polarized to an anti-inflammatory M2 phenotype displaying an impaired NF-κB activation, which may increase immunosuppressive capacity. Both IFN-γ or LPS can stimulate the classical pathway of M1 activation [71], but in our present study M1-related genes did not change significantly in PBEC-ALI/MQ models. Therefore, LPS contamination of the DEP used in the current study is an unlikely contributing factor, which was confirmed by the endotoxin levels measured using LAL-assay. These were all below detection limit. Interestingly, in co-cultured models, there were a high variability in cytokine/chemokine secretion after sham exposures, while after DEP exposures, the variation was smaller. Also, these phenomena were only observed in PBEC-ALI/MQ models. Because different phenotypes of the MQ release different cytokines, which may confirm that the proportions of different phenotypes of MQ were changed after DEP exposure compared to sham exposure. We assume that in sham exposure, the MQ in PBEC-ALI/MQ constituted a mixture of M0, M1 and M2 phenotypes with different proportions in each model. However, DEP exposure induced the MQ polarization to M2 phenotype, which induced a different secretion pattern with smaller variations in the MQ phenotypes of each individual model, and subsequently reduced the variability in the cytokine secretion.

One may speculate that the observed stimulation of M2-MQs related genes after an acute exposure to DEP could be a normal defense response following such exposures. Chronic exposure to DEP in contrast, may induce a M1-MQ response leading to a persistent inflammation, which may warrant further investigation.

## Conclusion

Taken together, we have demonstrated that DEP induced an inflammatory and oxidative stress response in the PBEC-ALI model, which were reduced in presence of MQ. This cell–cell interaction in the multicellular ALI-model (PBEC-ALI/MQ), in association with exposure to aerosolized ambient particles (DEP), allowed the direct interaction of particles with cell surfaces in a manner that better represented the in vivo situation. This cellular interaction of pulmonary epithelial cells with MQ in response to ambient particles played a pivotal role for MQ phenotypic alteration towards M2-subtypes, resulting in efficient resolution of the inflammatory response. This study also highlighted the fact that even mono-cell models cultured at ALI may be insufficient to investigate the detailed molecular responses, since cell-cell crosstalk is an important factor for the effects of air pollutant exposure. Finally, our physiologically relevant multi-cellular in vitro models in combination with the advanced exposure system (Xpose*ALI*^®^) has been shown to be effective in exposing ALI-models to dry aerosolized particles, mimicking the in vivo exposure situation to airborne pollutants.

## Additional file

Additional file 1: Supplement. **Table S1**. Primer Used for Quantitative Real-Time PCR (qPCR). **Figure S1**. Positive controls for inflammation, oxidative stress and M1/M2 polarization. **Figure S2**. Release and mRNA expression of inflammatory biomarkers after exposures to diesel exhaust particulates (DEPs). **Figure S3**. Cytotoxicity and cell viability assays to assess the effect of diesel exhaust particles (DEP) exposure in air-liquid interface models using lactate dehydrogenase assay (LDH) and apoptotic cell rate. **Figure S4**. The ratios of primary bronchial epithelial cells (PBEC) and THP-1 cell derived macrophages (MQ) in PBEC-ALI/MQ after exposure to diesel exhaust particulates (DEPs). **Figure S5**. mRNA expression of M1 macrophage markers after exposure to diesel exhaust particles (DEP). (ZIP 1014 kb)

## References

[1] Paulin L, Hansel N. Particulate air pollution and impaired lung function. F1000Res. 2016;5. PMID:26962445 10.12688/f1000research.7108.1 PMC4765726 26962445

[2] Salvi S, Blomberg A, Rudell B, Kelly F, Sandstrom T, Holgate ST, Frew A (1999). Acute inflammatory responses in the airways and peripheral blood after short-term exposure to diesel exhaust in healthy human volunteers. Am J Respir Crit Care Med.

[3] Mazzarella G, Ferraraccio F, Prati MV, Annunziata S, Bianco A, Mezzogiorno A, Liguori G, Angelillo IF, Cazzola M (2007). Effects of diesel exhaust particles on human lung epithelial cells: an in vitro study. Respir Med.

[4] Sehlstedt M, Behndig AF, Boman C, Blomberg A, Sandstrom T, Pourazar J (2010). Airway inflammatory response to diesel exhaust generated at urban cycle running conditions. Inhal Toxicol.

[5] Pronk A, Coble J, Stewart PA (2009). Occupational exposure to diesel engine exhaust: a literature review. J Expo Sci Environ Epidemiol.

[6] Schwarze PE, Totlandsdal AI, Lag M, Refsnes M, Holme JA, Ovrevik J (2013). Inflammation-related effects of diesel engine exhaust particles: studies on lung cells in vitro. Biomed Res Int.

[7] Silverman DT, Samanic CM, Lubin JH, Blair AE, Stewart PA, Vermeulen R, Coble JB, Rothman N, Schleiff PL, Travis WD (2012). The diesel exhaust in miners study: a nested case-control study of lung cancer and diesel exhaust. J Natl Cancer Inst.

[8] Thurston GD, Kipen H, Annesi-Maesano I, Balmes J, Brook RD, Cromar K, De Matteis S, Forastiere F, Forsberg B, Frampton MW, et al. A joint ERS/ATS policy statement: what constitutes an adverse health effect of air pollution? An analytical framework. Eur Respir J. 2017;49. PMID:28077473 10.1183/13993003.00419-2016 PMC5751718 28077473

[9] Yoshizaki K, Brito JM, Moriya HT, Toledo AC, Ferzilan S, Ligeiro de Oliveira AP, Machado ID, Farsky SH, Silva LF, Martins MA (2015). Chronic exposure of diesel exhaust particles induces alveolar enlargement in mice. Respir Res.

[10] Shvedova AA, Yanamala N, Murray AR, Kisin ER, Khaliullin T, Hatfield MK, Tkach AV, Krantz QT, Nash D, King C (2013). Oxidative stress, inflammatory biomarkers, and toxicity in mouse lung and liver after inhalation exposure to 100% biodiesel or petroleum diesel emissions. J Toxicol Environ Health A.

[11] Labranche N, Khattabi CE, Berkenboom G, Pochet S (2017). Effects of diesel exhaust particles on macrophage polarization. Hum Exp Toxicol.

[12] Tomasek I, Horwell CJ, Damby DE, Barosova H, Geers C, Petri-Fink A, Rothen-Rutishauser B, Clift MJ (2016). Combined exposure of diesel exhaust particles and respirable Soufriere Hills volcanic ash causes a (pro-)inflammatory response in an in vitro multicellular epithelial tissue barrier model. Part Fibre Toxicol.

[13] Chaudhuri N, Paiva C, Donaldson K, Duffin R, Parker LC, Sabroe I (2010). Diesel exhaust particles override natural injury-limiting pathways in the lung. Am J Physiol Lung Cell Mol Physiol.

[14] Risom L, Moller P, Loft S (2005). Oxidative stress-induced DNA damage by particulate air pollution. Mutat Res.

[15] Kaimul Ahsan M, Nakamura H, Tanito M, Yamada K, Utsumi H, Yodoi J (2005). Thioredoxin-1 suppresses lung injury and apoptosis induced by diesel exhaust particles (DEP) by scavenging reactive oxygen species and by inhibiting DEP-induced downregulation of Akt. Free Radic Biol Med.

[16] Poljsak B, Suput D, Milisav I (2013). Achieving the balance between ROS and antioxidants: when to use the synthetic antioxidants. Oxidative Med Cell Longev.

[17] Bauer RN, Muller L, Brighton LE, Duncan KE, Jaspers I (2015). Interaction with epithelial cells modifies airway macrophage response to ozone. Am J Respir Cell Mol Biol.

[18] Lohmann-Matthes ML, Steinmuller C, Franke-Ullmann G (1994). Pulmonary macrophages. Eur Respir J.

[19] Proud D, Leigh R (2011). Epithelial cells and airway diseases. Immunol Rev.

[20] Biswas SK, Mantovani A (2010). Macrophage plasticity and interaction with lymphocyte subsets: cancer as a paradigm. Nat Immunol.

[21] Mosser DM, Edwards JP (2008). Exploring the full spectrum of macrophage activation. Nat Rev Immunol.

[22] Boorsma CE, Draijer C, Melgert BN (2013). Macrophage heterogeneity in respiratory diseases. Mediat Inflamm.

[23] Martinez FO, Helming L, Milde R, Varin A, Melgert BN, Draijer C, Thomas B, Fabbri M, Crawshaw A, Ho LP (2013). Genetic programs expressed in resting and IL-4 alternatively activated mouse and human macrophages: similarities and differences. Blood.

[24] Byrne AJ, Mathie SA, Gregory LG, Lloyd CM (2015). Pulmonary macrophages: key players in the innate defence of the airways. Thorax.

[25] Shaykhiev R, Krause A, Salit J, Strulovici-Barel Y, Harvey BG, O'Connor TP, Crystal RG (2009). Smoking-dependent reprogramming of alveolar macrophage polarization: implication for pathogenesis of chronic obstructive pulmonary disease. J Immunol.

[26] Lucarelli M, Gatti AM, Savarino G, Quattroni P, Martinelli L, Monari E, Boraschi D (2004). Innate defence functions of macrophages can be biased by nano-sized ceramic and metallic particles. Eur Cytokine Netw.

[27] Yen HJ, Hsu SH, Tsai CL (2009). Cytotoxicity and immunological response of gold and silver nanoparticles of different sizes. Small.

[28] Tran TH, Rastogi R, Shelke J, Amiji MM (2015). Modulation of macrophage functional polarity towards anti-inflammatory phenotype with plasmid DNA delivery in CD44 targeting hyaluronic acid nanoparticles. Sci Rep.

[29] Miao X, Leng X, Zhang Q. The current state of nanoparticle-induced macrophage polarization and reprogramming research. Int J Mol Sci. 2017;18. PMID:28178185 10.3390/ijms18020336 PMC5343871 28178185

[30] Jaguin M, Fardel O, Lecureur V (2015). Exposure to diesel exhaust particle extracts (DEPe) impairs some polarization markers and functions of human macrophages through activation of AhR and Nrf2. PLoS One.

[31] Sawyer K, Mundandhara S, Ghio AJ, Madden MC (2010). The effects of ambient particulate matter on human alveolar macrophage oxidative and inflammatory responses. J Toxicol Environ Health A.

[32] Sunil VR, Patel-Vayas K, Shen J, Laskin JD, Laskin DL (2012). Classical and alternative macrophage activation in the lung following ozone-induced oxidative stress. Toxicol Appl Pharmacol.

[33] Guth AM, Janssen WJ, Bosio CM, Crouch EC, Henson PM, Dow SW (2009). Lung environment determines unique phenotype of alveolar macrophages. Am J Physiol Lung Cell Mol Physiol.

[34] Miyata R, van Eeden SF (2011). The innate and adaptive immune response induced by alveolar macrophages exposed to ambient particulate matter. Toxicol Appl Pharmacol.

[35] Ji J, von Scheele I, Billing B, Dahlen B, Lantz AS, Larsson K, Palmberg L (2016). Effects of budesonide on toll-like receptor expression in alveolar macrophages from smokers with and without COPD. Int J Chron Obstruct Pulmon Dis.

[36] von Scheele I, Larsson K, Palmberg L (2010). Budesonide enhances toll-like receptor 2 expression in activated bronchial epithelial cells. Inhal Toxicol.

[37] Mundandhara SD, Becker S, Madden MC (2006). Effects of diesel exhaust particles on human alveolar macrophage ability to secrete inflammatory mediators in response to lipopolysaccharide. Toxicol in Vitro.

[38] Strandberg K, Palmberg L, Larsson K (2008). Effect of budesonide and formoterol on IL-6 and IL-8 release from primary bronchial epithelial cells. J Asthma.

[39] Ji J, Hedelin A, Malmlof M, Kessler V, Seisenbaeva G, Gerde P, Palmberg L (2017). Development of combining of human bronchial mucosa models with XposeALI(R) for exposure of air pollution nanoparticles. PLoS One.

[40] Auwerx J (1991). The human leukemia cell line, THP-1: a multifacetted model for the study of monocyte-macrophage differentiation. Experientia.

[41] Park EK, Jung HS, Yang HI, Yoo MC, Kim C, Kim KS (2007). Optimized THP-1 differentiation is required for the detection of responses to weak stimuli. Inflamm Res.

[42] Wottrich R, Diabate S, Krug HF (2004). Biological effects of ultrafine model particles in human macrophages and epithelial cells in mono- and co-culture. Int J Hyg Environ Health.

[43] Rudd CJ, Strom KA (1981). A spectrophotometric method for the quantitation of diesel exhaust particles in Guinea pig lung. J Appl Toxicol.

[44] Dwivedi AM, Upadhyay S, Johanson G, Ernstgard L, Palmberg L (2018). Inflammatory effects of acrolein, crotonaldehyde and hexanal vapors on human primary bronchial epithelial cells cultured at air-liquid interface. Toxicol in Vitro.

[45] Larsson K, Tornling G, Gavhed D, Muller-Suur C, Palmberg L (1998). Inhalation of cold air increases the number of inflammatory cells in the lungs in healthy subjects. Eur Respir J.

[46] Maecker HT, Trotter J (2006). Flow cytometry controls, instrument setup, and the determination of positivity. Cytometry A.

[47] Kireeva ED, Popovicheva OB, Persiantseva NM, Timofeyev MA, Shonija NK (2009). Fractionation analysis of transport engine-generated soot particles with respect to hygroscopicity. J Atmos Chem.

[48] Franks TJ, Colby TV, Travis WD, Tuder RM, Reynolds HY, Brody AR, Cardoso WV, Crystal RG, Drake CJ, Engelhardt J (2008). Resident cellular components of the human lung: current knowledge and goals for research on cell phenotyping and function. Proc Am Thorac Soc.

[49] Joris F, Manshian BB, Peynshaert K, De Smedt SC, Braeckmans K, Soenen SJ (2013). Assessing nanoparticle toxicity in cell-based assays: influence of cell culture parameters and optimized models for bridging the in vitro-in vivo gap. Chem Soc Rev.

[50] Paur HR, Cassee FR, Teeguarden J, Fissan H, Diabate S, Aufderheide M, Kreyling WG, Hanninen O, Kasper G, Riediker M (2011). In-vitro cell exposure studies for the assessment of nanoparticle toxicity in the lung-a dialog between aerosol science and biology. J Aerosol Sci.

[51] Lenz AG, Karg E, Brendel E, Hinze-Heyn H, Maier KL, Eickelberg O, Stoeger T, Schmid O (2013). Inflammatory and oxidative stress responses of an alveolar epithelial cell line to airborne zinc oxide nanoparticles at the air-liquid interface: a comparison with conventional, submerged cell-culture conditions. Biomed Res Int.

[52] Limbach LK, Li Y, Grass RN, Brunner TJ, Hintermann MA, Muller M, Gunther D, Stark WJ (2005). Oxide nanoparticle uptake in human lung fibroblasts: effects of particle size, agglomeration, and diffusion at low concentrations. Environ Sci Technol.

[53] Ji J, von Scheele I, Bergstrom J, Billing B, Dahlen B, Lantz AS, Larsson K, Palmberg L (2014). Compartment differences of inflammatory activity in chronic obstructive pulmonary disease. Respir Res.

[54] Takizawa H, Ohtoshi T, Kawasaki S, Kohyama T, Desaki M, Kasama T, Kobayashi K, Nakahara K, Yamamoto K, Matsushima K, Kudoh S (1999). Diesel exhaust particles induce NF-kappa B activation in human bronchial epithelial cells in vitro: importance in cytokine transcription. J Immunol.

[55] Steerenberg PA, Zonnenberg JA, Dormans JA, Joon PN, Wouters IM, van Bree L, Scheepers PT, Van Loveren H (1998). Diesel exhaust particles induced release of interleukin 6 and 8 by (primed) human bronchial epithelial cells (BEAS 2B) in vitro. Exp Lung Res.

[56] Ball JC, Straccia AM, Young WC, Aust AE (2000). The formation of reactive oxygen species catalyzed by neutral, aqueous extracts of NIST ambient particulate matter and diesel engine particles. J Air Waste Manag Assoc.

[57] Kaspar JW, Niture SK, Jaiswal AK (2009). Nrf2:INrf2 (Keap1) signaling in oxidative stress. Free Radic Biol Med.

[58] Motohashi H, Yamamoto M (2004). Nrf2-Keap1 defines a physiologically important stress response mechanism. Trends Mol Med.

[59] Zarcone MC, van Schadewijk A, Duistermaat E, Hiemstra PS, Kooter IM (2017). Diesel exhaust alters the response of cultured primary bronchial epithelial cells from patients with chronic obstructive pulmonary disease (COPD) to non-typeable Haemophilus influenzae. Respir Res.

[60] Ishii H, Hayashi S, Hogg JC, Fujii T, Goto Y, Sakamoto N, Mukae H, Vincent R, van Eeden SF (2005). Alveolar macrophage-epithelial cell interaction following exposure to atmospheric particles induces the release of mediators involved in monocyte mobilization and recruitment. Respir Res.

[61] Doornaert B, Leblond V, Galiacy S, Gras G, Planus E, Laurent V, Isabey D, Lafuma C (2003). Negative impact of DEP exposure on human airway epithelial cell adhesion, stiffness, and repair. Am J Physiol Lung Cell Mol Physiol.

[62] Amara N, Bachoual R, Desmard M, Golda S, Guichard C, Lanone S, Aubier M, Ogier-Denis E, Boczkowski J (2007). Diesel exhaust particles induce matrix metalloprotease-1 in human lung epithelial cells via a NADP(H) oxidase/NOX4 redox-dependent mechanism. Am J Physiol Lung Cell Mol Physiol.

[63] Leema G, Swapna U, Koustav G, Tobias S. Macrophage Polarization in Lung Biology and Diseases. In Lung Inflammation. Edited by (Ed.) DK-CO. London: InTech; 2014.

[64] Becker S, Fenton MJ, Soukup JM (2002). Involvement of microbial components and toll-like receptors 2 and 4 in cytokine responses to air pollution particles. Am J Respir Cell Mol Biol.

[65] Becker S, Dailey L, Soukup JM, Silbajoris R, Devlin RB (2005). TLR-2 is involved in airway epithelial cell response to air pollution particles. Toxicol Appl Pharmacol.

[66] Williams MA, Porter M, Horton M, Guo J, Roman J, Williams D, Breysse P, Georas SN (2007). Ambient particulate matter directs nonclassic dendritic cell activation and a mixed TH1/TH2-like cytokine response by naive CD4+ T cells. J Allergy Clin Immunol.

[67] Kawasaki T, Kawai T (2014). Toll-like receptor signaling pathways. Front Immunol.

[68] Mayer AK, Bartz H, Fey F, Schmidt LM, Dalpke AH (2008). Airway epithelial cells modify immune responses by inducing an anti-inflammatory microenvironment. Eur J Immunol.

[69] Martinez FO, Gordon S (2014). The M1 and M2 paradigm of macrophage activation: time for reassessment. F1000Prime Rep.

[70] Mantovani A, Sica A, Sozzani S, Allavena P, Vecchi A, Locati M (2004). The chemokine system in diverse forms of macrophage activation and polarization. Trends Immunol.

[71] Wang N, Liang H, Zen K (2014). Molecular mechanisms that influence the macrophage m1-m2 polarization balance. Front Immunol.

[72] Sauer RS, Hackel D, Morschel L, Sahlbach H, Wang Y, Mousa SA, Roewer N, Brack A, Rittner HL (2014). Toll like receptor (TLR)-4 as a regulator of peripheral endogenous opioid-mediated analgesia in inflammation. Mol Pain.

[73] Orr JS, Puglisi MJ, Ellacott KL, Lumeng CN, Wasserman DH, Hasty AH (2012). Toll-like receptor 4 deficiency promotes the alternative activation of adipose tissue macrophages. Diabetes.

[74] Schlaepfer E, Rochat MA, Duo L, Speck RF (2014). Triggering TLR2, −3, −4, −5, and −8 reinforces the restrictive nature of M1- and M2-polarized macrophages to HIV. J Virol.

