# Supplementary material for: Multi-cellular human bronchial models exposed to diesel exhaust particles: assessment of inflammation, oxidative stress and macrophage polarization
Source: Part Fibre Toxicol. 2018 May 2;15:19. doi: 10.1186/s12989-018-0256-2 (PMC5930819; doi:10.1186/s12989-018-0256-2)
Supplement: Supplementary file 1 — Supplement. Table S1. Primer Used for Quantitative Real-Time PCR (qPCR). Figure S1. Positive controls for inflammation, oxidative stress and M1/M2 polarization. Figure S2. Release and mRNA expression of inflammatory biomarkers after exposures to diesel exhaust particulates (DEPs). Figure S3. Cytotoxicity and cell viability assays to assess the effect of diesel exhaust particles (DEP) exposure in air-liquid interface models using lactate dehydrogenase assay (LDH) and apoptotic cell rate. Figure S4. The ratios of primary bronchial epithelial cells (PBEC) and THP-1 cell derived macrophages (MQ) in PBEC-ALI/MQ after exposure to diesel exhaust particulates (DEPs). Figure S5. mRNA expression of M1 macrophage markers after exposure to diesel exhaust particles (DEP). (ZIP 1014 kb) [file 12989_2018_256_MOESM1_ESM.zip › supplement_track change.docx]

**Supplement of: Multi-cellular Human Bronchial Models Exposed to Diesel Exhaust Particles: Assessment of Inflammation, Oxidative Stress and Macrophage Polarization**

In this study, we have cultured primary bronchial epithelial cells (PBEC) at air-liquid interface (ALI) which were co-cultured without (PBEC-ALI) and with macrophages (PBEC-ALI/MQ) models. For the co-cultured models, two different settings were tested. Firstly, we co-cultured MQ placed underneath the PBEC-ALI models (PBEC-ALI/MQ_sub_), without having any direct contact between MQ and PBEC-ALI. Secondly PBEC-ALI was co-cultured with MQ on top of the epithelial layer (PBEC-ALI/MQ), and this latter model was chosen for all other studies as described in the main manuscript.

The establishment of PBEC-ALI models were the same as described in the method for PBEC-ALI/MQ_sub_. The DEP exposures were carried out using Xpose*ALI*^®^ for 15s (low), 45s (medium) and 3 mins (high). Spectrophotometric technique was used to detect absorbance of DEP in 99% ethanol [1]. The DEP standard curve was built by the absorbance of 7 known concentrations of the DEP in 99% ethanol solutions (1.56, 3.13, 6.25,12.5, 25, 50, 100µg/ml). For each time duration, three DEP exposed inserts were rinsed with 200µl 99% ethanol. The absorbance was detected and the DEP dose was back calculated based on DEP standard curve. The average dose of three inserts was identified as the dose of each exposure dose. The DEP dose used corresponded to 3.4, 5 and 12.7 µg/cm^2^ respectively for the low-, medium- and high exposure doses. Different doses of DEP exposure were performed to identify the most effective dose. Control (sham) models were exposed to clean air. Following exposure, DEP and sham exposed PBEC-ALI models were transferred to 12-wells-plates which were pre-cultured with MQ. The cultured MQ was in the well just below each insert, without having any direct contact with PBEC. We continued to co-culture the exposed models for 6 and 24 hours in 5% CO_2_ and 37ºC. The secretion of CXCL-8 and CC10 in basal medium was detected by ELISA. To detect mRNA expression of *CXCL8, TNFα, IL6, HMOX1* and *NFKB*, the PBEC model was taken out from the 12-wells-plats after 24 hours of incubation, trypsinized and qRT-PCR were performed. Cell viability was tested by using trypan blue and LDH-assay. FACS was used to identify apoptosis rate as described in method part. In the pilot studies, we treated different models with LPS and H_2_O_2_ as positive controls to induce inflammation and oxidative stress, respectively. We found that the secretion of CXCL-8and TNF-α were increased in 24hours H_2_O_2_ (100µM) or LPS (1µg/ml) treated groups compare to untreated groups (Additional file 1: Figure S1A&B). Treatment of THP-1 derived macrophages with LPS (10ng/ml) + IFNγ (20ng/ml) and IL-4 (20ng/ml) for 24hous were also used as positive controls for M1 and M2 macrophages[1] (Additional file 1: Figure S1C-F). The mRNA expression of M1 and M2 macrophage markers were increased accordingly after the stimulations.

Additional file 1: Figure S2 showed the release of CXCL-8 in basal medium. The highest doses of DEP increased CXCL-8 secretion after 6 h incubation (Additional file 1: Figure S2 A). Both PBEC-ALI and PBEC-ALI/MQ_sub_ secreted significantly more CXCL-8 and CC10 after 24 h compared to 6 h except after exposure of medium dose of DEP (Additional file 1: Figure S2 A, data not shown). However, mRNA expression of *CXCL8*, IL6, and *TNFα* were more in PBEC-ALI/MQ_sub_ compared with in PBEC-ALI following 6 h to DEP (Additional file 1: Figure S2 B, C, and D). The expression of *CXCL8* in PBEC-ALI increased significantly after 24 h exposure to low dose of DEP (Additional file 1: Figure S2 B). Expression of *TNFα* increased significantly at low DEP dose exposure followed by 6h incubation in PBEC-ALI and after 24 h in PBEC-ALI/MQ_sub_ (Figure 2 D). High dose DEP significantly enhanced the expression of *CXCL8* and *TNFα* after 6h incubation in both PBEC-ALI and co-cultured models (Additional file 1: Figure S2 B, D). Similarly, high dose DEP exposure increased *CXCL8* expression in PBEC-ALI and *TNFα* in co-cultured models after 24h incubation (Additional file 1: Figure S2 B, D). However, such responses were not observed in protein level (CC10) or mRNA expression (*CC10*, *HMOX1* and *NFKB)* following DEP exposure and/or the presence of MQ (data not shown).

The results above showed that the PBEC-ALI/MQ_sub_ did not respond to DEP exposure. According to Striz´s study [2], cell-cell contacts between epithelial cells and MQ was very critical in the regulation of macrophage phenotype. Further, *in vivo*, macrophages are in direct contact with epithelial cell surface which facilitate interaction with bronchial epithelial cells [3], Therefore it is more physiologically relevant to have MQ and PBEC in direct contact with each other. After confirmation that MQ survive when cultured at ALI with no cell culture media on top. We chose to develop models with the MQ on top of the epithelial cell layers (PBEC-ALI/MQ), where MQ were added on the apical side of the PBEC-ALI with mucus present.

Further, based on the results from pilot study described above, we found that the highest dose of DEP exposure (12.7 µg/cm^2^) could significantly stimulate the models without affecting cell viability during an incubation time of 24 hours which, is an optimal time to cover the total cell cycle for proliferation of human cells [4]. Hence, we have performed a further detailed study on assessment of inflammation, oxidative stress and macrophage polarization following exposure of both PBEC-ALI and (PBEC-ALI/MQ), to aerosolized DEP (12.7 µg/cm^2^) as described in the main manuscript.

**Table S1:** Primer Used for Quantitative Real-Time PCR (qPCR)

| **Gene name** | **Gene Symbol** | **Forward Primer 5’-3’** | **Reverse Primer 3’-5’** |
| --- | --- | --- | --- |
| Beta (β)-Actin | ACTB | CTGGGACGACATGCAGAAAA | AAGGAAGGCTGGAAGAGTGC |
| Nuclear factor kappa-light-chain-enhancer of activated B cells | NFKB | AAGAGGAGGTTTCGCCACCG | TTGCAGATTTTGACCTGAGGGT |
| Tumor necrosis factor alpha | TNFα | AGCCCATGTTGTAGCAAACC | ACATTGGGTCCCCCAGGATA |
| Interleukin 6 | IL6 | ACCCCCAGGAGAAGATTCCA | CACCAGGCAAGTCTCCTCATT |
| C-X-C Motif Chemokine Ligand 8 | CXCL8 | GCTCTGTGTGAAGGTGCAGTT | GGCACAGTGGAACAAGGACT |
| Toll-like receptor 2 | TLR2 | CTCATTGTGCCCATTGCTCTT | TCCAGTGCTTCAACCCACAAC |
| Toll-like receptor 4 | TLR4 | GGCCATTGCTGCCAACAT | CAACAATCACCTTTCGGCTTTT |
| Matrix metallopeptidase 9 | MMP9 | GGTGATTGACGACGCCTTTG | GGACCACAACTCGTCATCGT |
| Tissue inhibitor of metalloproteinases 1 | TIMP1 | ATTTATCCTCTAGCGCTCAGGCCC | AATTGCAGAAGGCCGTCTGTGG |
| Heme oxygenase 1 | HMOX1 | TTCAAGCAGCTCTACCGCTC | GGGGGCAGAATCTTGCACTTT |
| Glutathione Peroxidase 1 | GPX1 | AGTCGGTGTATGCCTTCTCGGC | CGTTCTCCTGATGCCCAAACTG |
| Interleukin 10 | IL10 | GAGAACAGCTGCACCCACTT | TTCTCAGCTTGGGGCATCAC |
| Interleukin 4 | IL4 | GCACCGAGTTGACCGTAACA | CCAACGTACTCTGGTTGGCT |
| Mannose Receptor C-Type 1 | MRC1 | CTGAATTGTACTGGTCTGTCCT | GCTTAGATGTGGTGCTGTGG |
| Mannose Receptor C-Type 2 | MRC2 | CTACCTCAACGGCACCTTCC | TCTGAGACCCAGGAGTACCG |
| Resistin Like Beta | RETNLB | GCTCTCGTGTGCTAGTGTCA | GGTTGGGACCCTGGTTTCAT |

**Supplementary Figure Legends**

**Figure S1: Positive controls for inflammation, oxidative stress and M1/M2 polarization.**

Levels of CXCL-8 (1A) and TNF-α (1B) secretions in PBEC-ALI (N=3) treated with H_2_O_2_ (100µM) or LPS (1µg/ml) for 24hours; Fold change of *IL12* (1C), *MRC1* (1D), *MRC2* (1E) and *RETNLA* (1H) expression in THP-1 derived macrophages (N=2) treated with LPS (10ng/ml) + IFNγ (20ng/ml) (M1-macrophage) and IL-4 (20ng/ml) (M2-macrophage) for 24hous. Data presented as median and 25^th^ -75^th^ percentiles, fold change =2^-ΔCt^ of treated THP-1 derived macrophages / 2^-ΔCt^ of untreated THP-1 derived macrophages.

**Figure S2: Release and mRNA expression of inflammatory biomarkers after exposures to diesel exhaust particulates (DEPs).**

Levels of CXCL-8 (2A) secretion in basal medium in PBEC-ALI and co-culture models with MQ underneath models (N=9) after exposure to DEPs and incubated for 6 and 24 hours; Fold change of *CXCL8* (2B), *TNFα* (2C) and *IL6*( 2D) expression in PBEC-ALI and co-cultured models (N=6) after exposure to Sham or DEPs and incubated for 6 and 24 hours; Exposure: sham: clean air; DEPs: Low:3.4µg/cm^2^, Med:5.0 µg/cm^2^, High:12.7 µg/cm^2^; Data presented as median and 25^th^ -75^th^ percentiles, fold change =2^-ΔCt^ of models / 2^-ΔCt^ of sham exposed PBEC-ALI; *,**: P<0.05, 0.01 VS Sham exposure; #: P<0.05 VS PBEC-ALI; &,&&: P<0.05, 0.01 VS 6 hours.

**Figure S3: Cytotoxicity and cell viability assays to assess the effect of diesel exhaust particles (DEP) exposure in air-liquid interface models using lactate dehydrogenase assay (LDH) and apoptotic cell rate.**

3A: The colorimetric LDH assay to measure the cytotoxic effect of DEP in PBEC-ALI and PBEC-ALI/MQ (N=3) 24 hours post exposure to aerosolized DEP compare to Sham, 1% Titron X and positive control (provided in the Kit). 3B: The cellular apoptosis rates of PBEC-ALI and PBEC-ALI/MQ (N=3) after exposure to DEP and incubated for 24 hours; The detections were performed by FACS with annexin V–PE/7-AAD. Exposure: sham: clean air; DEP: 12.7µg/cm^2^; Data presented as median and 25^th^ -75^th^ percentiles, total apoptosis rate= early apoptosis rate +late apoptosis rate.

**Figure S4: The ratios of primary bronchial epithelial cells (PBEC) and THP-1 cell derived macrophages (MQ) in PBEC-ALI/MQ after exposure to diesel exhaust particulates (DEPs).**

PBEC and MQ were identified by anti-CD 68-PE-Cy7. The CD68 ^+^ cells were MQ and CD68 ^-^ cells were PBEC. A representative of 9 experiments is shown. Numbers in figure indicate percentage of cells in each gate: CD68^-^ cell (PBEC) is 80% and CD68^+^(MQ) cell is 8.1%.

**Figure S5: mRNA expression of M1 macrophage markers after exposure to diesel exhaust particles (DEP).**

Fold change of *IL12* (5A) and *IL23* (5B) expression in PBEC-ALI and PBEC-AL/MQ (N=6) after exposure to DEP and incubated for 24 hours; Exposure: sham: clean air; DEP: 12.7µg/cm^2^; Data presented as median and 25^th^ -75^th^ percentiles, fold change =2^-ΔCt^ of models / 2^-ΔCt^ of sham exposed PBEC-ALI.

**References**

1. Genin, M., et al., *M1 and M2 macrophages derived from THP-1 cells differentially modulate the response of cancer cells to etoposide.* BMC Cancer, 2015. **15**: p. 577.

2. Striz, I., et al., *Cell-cell contacts with epithelial cells modulate the phenotype of human macrophages.* Inflammation, 2001. **25**(4): p. 241-6.

3. Lay, J.C., D.B. Peden, and N.E. Alexis, *Flow cytometry of sputum: assessing inflammation and immune response elements in the bronchial airways.* Inhal Toxicol, 2011. **23**(7): p. 392-406.

4. Cooper, G.M. and National Center for Biotechnology Information (U.S.), *The cell a molecular approach*. 2000, ASM Press ;Sinauer Associates,: Washington, D.C. Sunderland, Mass.
