# Supplementary figures and images for: Multi-cellular human bronchial models exposed to diesel exhaust particles: assessment of inflammation, oxidative stress and macrophage polarization
Source: Part Fibre Toxicol. 2018 May 2;15:19. doi: 10.1186/s12989-018-0256-2 (PMC5930819; doi:10.1186/s12989-018-0256-2)

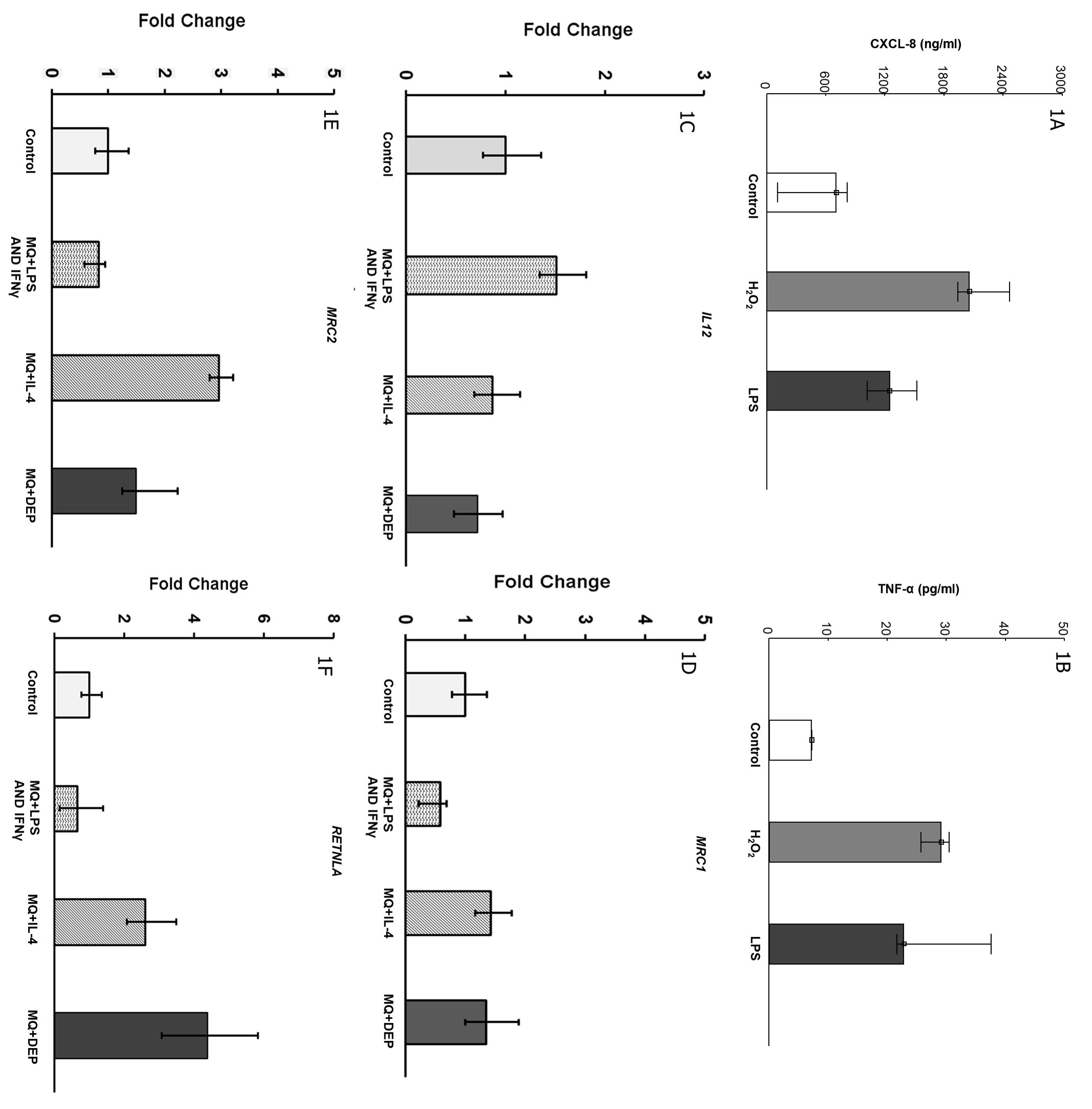

Supplement: Supplementary file 1 — Supplement. Table S1. Primer Used for Quantitative Real-Time PCR (qPCR). Figure S1. Positive controls for inflammation, oxidative stress and M1/M2 polarization. Figure S2. Release and mRNA expression of inflammatory biomarkers after exposures to diesel exhaust particulates (DEPs). Figure S3. Cytotoxicity and cell viability assays to assess the effect of diesel exhaust particles (DEP) exposure in air-liquid interface models using lactate dehydrogenase assay (LDH) and apoptotic cell rate. Figure S4. The ratios of primary bronchial epithelial cells (PBEC) and THP-1 cell derived macrophages (MQ) in PBEC-ALI/MQ after exposure to diesel exhaust particulates (DEPs). Figure S5. mRNA expression of M1 macrophage markers after exposure to diesel exhaust particles (DEP). (ZIP 1014 kb) [file 12989_2018_256_MOESM1_ESM.zip › SFigure 1.jpg]

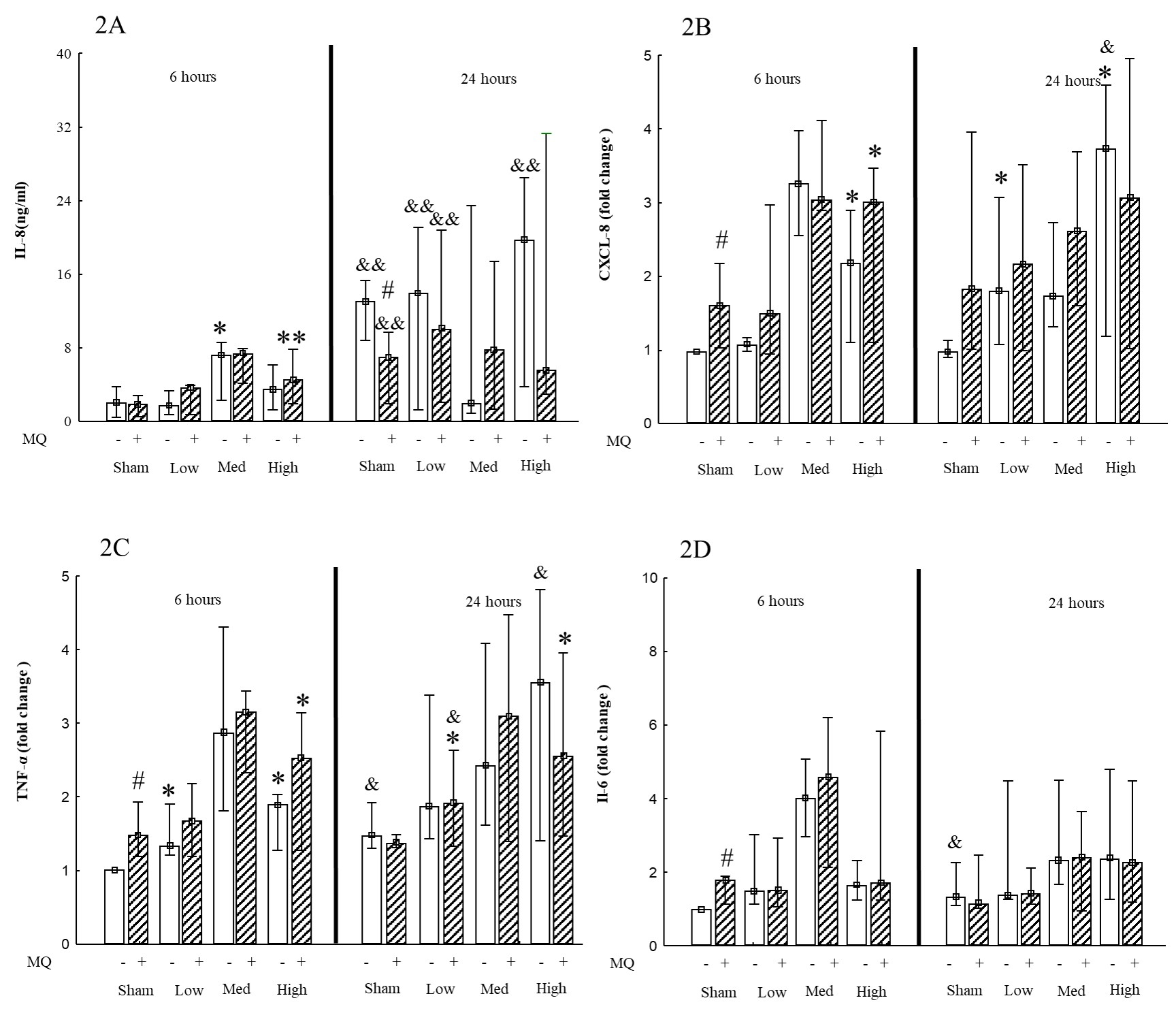

Supplement: Supplementary file 1 — Supplement. Table S1. Primer Used for Quantitative Real-Time PCR (qPCR). Figure S1. Positive controls for inflammation, oxidative stress and M1/M2 polarization. Figure S2. Release and mRNA expression of inflammatory biomarkers after exposures to diesel exhaust particulates (DEPs). Figure S3. Cytotoxicity and cell viability assays to assess the effect of diesel exhaust particles (DEP) exposure in air-liquid interface models using lactate dehydrogenase assay (LDH) and apoptotic cell rate. Figure S4. The ratios of primary bronchial epithelial cells (PBEC) and THP-1 cell derived macrophages (MQ) in PBEC-ALI/MQ after exposure to diesel exhaust particulates (DEPs). Figure S5. mRNA expression of M1 macrophage markers after exposure to diesel exhaust particles (DEP). (ZIP 1014 kb) [file 12989_2018_256_MOESM1_ESM.zip › SFigure 2.jpg]

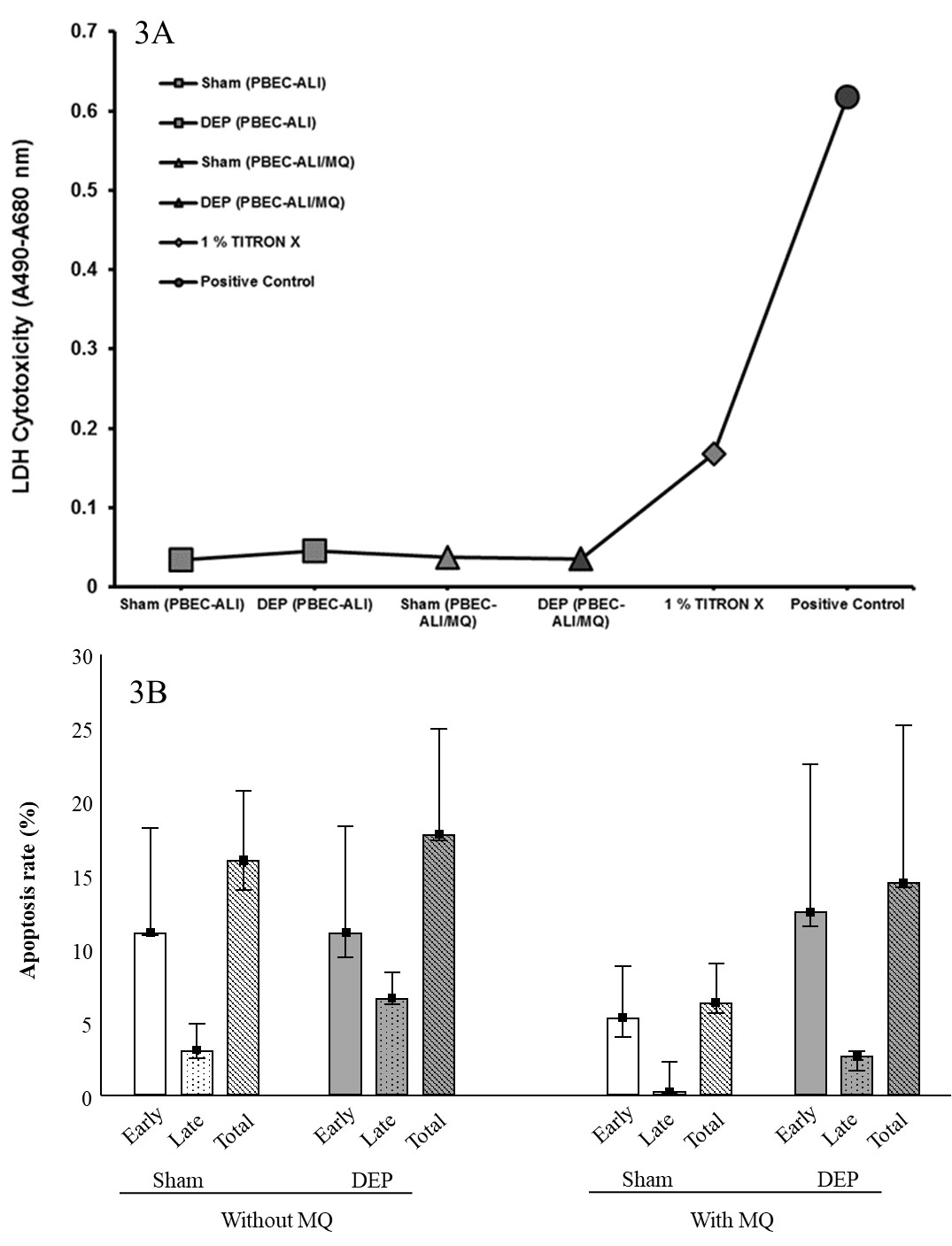

Supplement: Supplementary file 1 — Supplement. Table S1. Primer Used for Quantitative Real-Time PCR (qPCR). Figure S1. Positive controls for inflammation, oxidative stress and M1/M2 polarization. Figure S2. Release and mRNA expression of inflammatory biomarkers after exposures to diesel exhaust particulates (DEPs). Figure S3. Cytotoxicity and cell viability assays to assess the effect of diesel exhaust particles (DEP) exposure in air-liquid interface models using lactate dehydrogenase assay (LDH) and apoptotic cell rate. Figure S4. The ratios of primary bronchial epithelial cells (PBEC) and THP-1 cell derived macrophages (MQ) in PBEC-ALI/MQ after exposure to diesel exhaust particulates (DEPs). Figure S5. mRNA expression of M1 macrophage markers after exposure to diesel exhaust particles (DEP). (ZIP 1014 kb) [file 12989_2018_256_MOESM1_ESM.zip › SFigure 3.jpg]

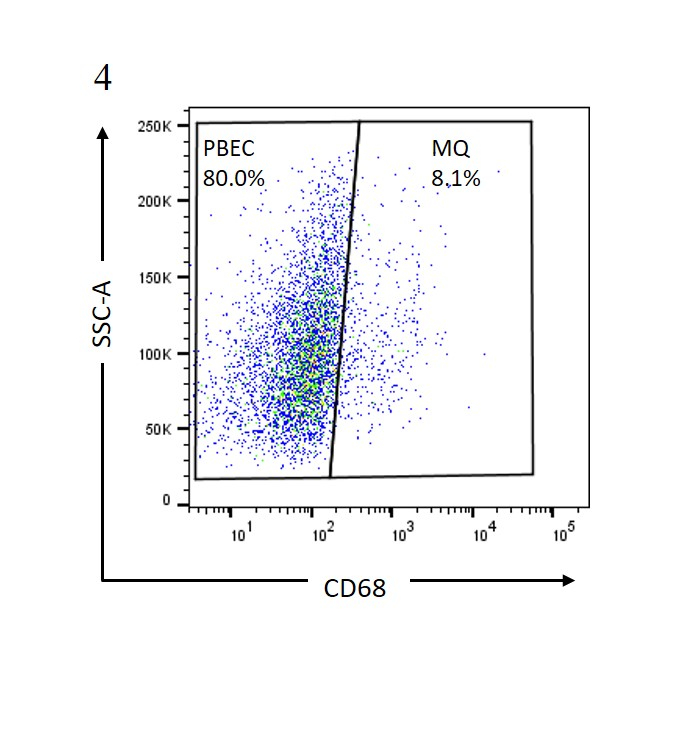

Supplement: Supplementary file 1 — Supplement. Table S1. Primer Used for Quantitative Real-Time PCR (qPCR). Figure S1. Positive controls for inflammation, oxidative stress and M1/M2 polarization. Figure S2. Release and mRNA expression of inflammatory biomarkers after exposures to diesel exhaust particulates (DEPs). Figure S3. Cytotoxicity and cell viability assays to assess the effect of diesel exhaust particles (DEP) exposure in air-liquid interface models using lactate dehydrogenase assay (LDH) and apoptotic cell rate. Figure S4. The ratios of primary bronchial epithelial cells (PBEC) and THP-1 cell derived macrophages (MQ) in PBEC-ALI/MQ after exposure to diesel exhaust particulates (DEPs). Figure S5. mRNA expression of M1 macrophage markers after exposure to diesel exhaust particles (DEP). (ZIP 1014 kb) [file 12989_2018_256_MOESM1_ESM.zip › SFigure 4.jpg]

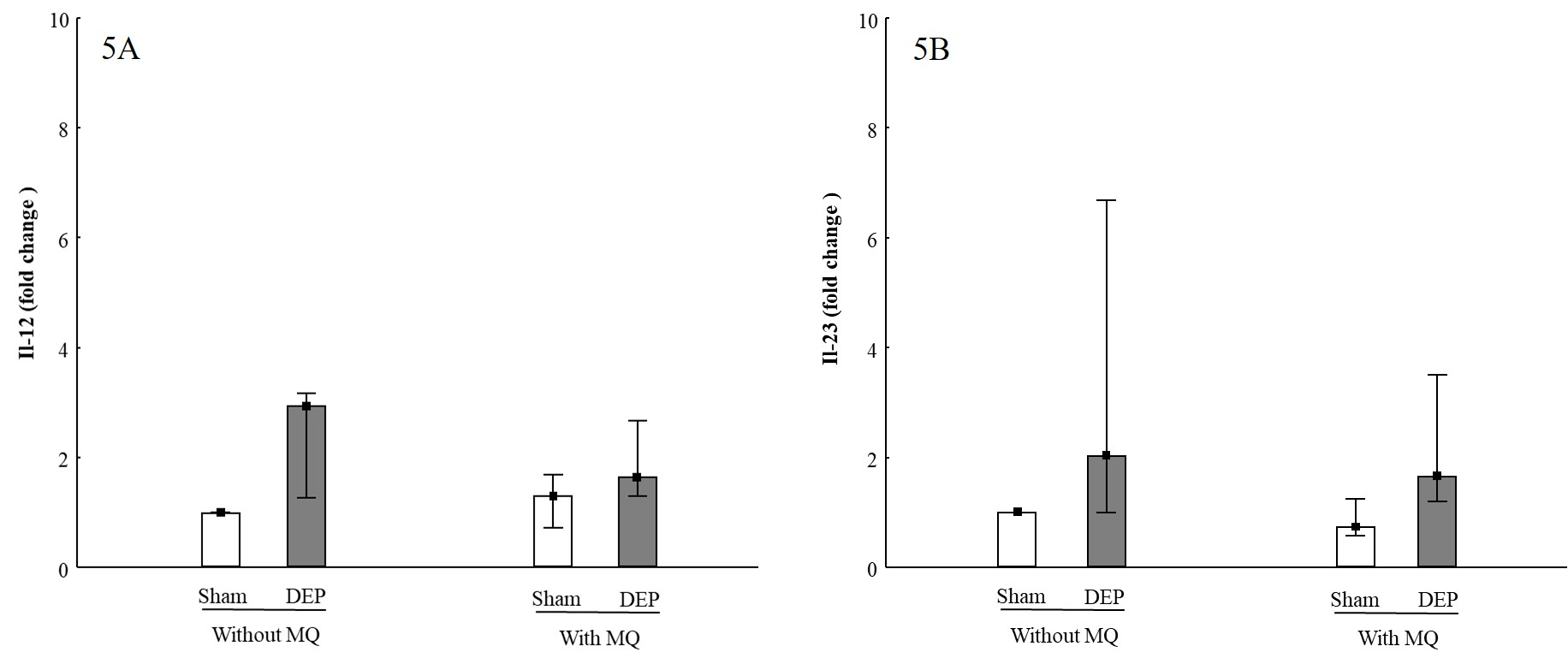

Supplement: Supplementary file 1 — Supplement. Table S1. Primer Used for Quantitative Real-Time PCR (qPCR). Figure S1. Positive controls for inflammation, oxidative stress and M1/M2 polarization. Figure S2. Release and mRNA expression of inflammatory biomarkers after exposures to diesel exhaust particulates (DEPs). Figure S3. Cytotoxicity and cell viability assays to assess the effect of diesel exhaust particles (DEP) exposure in air-liquid interface models using lactate dehydrogenase assay (LDH) and apoptotic cell rate. Figure S4. The ratios of primary bronchial epithelial cells (PBEC) and THP-1 cell derived macrophages (MQ) in PBEC-ALI/MQ after exposure to diesel exhaust particulates (DEPs). Figure S5. mRNA expression of M1 macrophage markers after exposure to diesel exhaust particles (DEP). (ZIP 1014 kb) [file 12989_2018_256_MOESM1_ESM.zip › SFigure 5.jpg]
